# When Helping is Risky: The Behavioral and Neurobiological Tradeoff of Social and Risk Preferences

**DOI:** 10.1177/09567976211015942

**Published:** 2021-10-27

**Authors:** Jörg Gross, Nadira S. Faber, Andreas Kappes, Anne-Marie Nussberger, Philip J Cowen, Michael Browning, Guy Kahane, Julian Savulescu, Molly J. Crockett, Carsten K.W. De Dreu

**Affiliations:** 1 Institute of Psychology, Leiden University; 2 College of Life and Environmental Sciences, University of Exeter; 3 Oxford Uehiro Centre for Practical Ethics, University of Oxford; 4 Department of Psychology, City, University of London; 5 Department of Experimental Psychology, University of Oxford; 6 Department of Psychiatry, University of Oxford; 7 Oxford Health NHS Foundation Trust, Oxford; 8 Department of Psychology, Yale University; 9 Center for Research in Experimental Economics and Political Decision Making (CREED), University of Amsterdam

## Abstract

Helping others can entail risks for the helper. For example, when treating infectious patients, medical volunteers risk their own health. In such situations, helping-decisions should depend on the individual’s valuation of others’ well-being (social preferences) and the degree of personal risk the individual finds acceptable (risk preferences). We investigate how these distinct preferences are psychologically and neurobiologically integrated when helping is risky. We used incentivized decision-making tasks (Study 1, N=292, mean age=22.3±3.7, 142 female) and manipulated dopamine and norepinephrine levels in the brain by administering methylphenidate, atomoxetine, or placebo (Study 2, N=154, mean age=23.7±3.9, 77 female). We find that social and risk preferences are independent drivers of risky helping. Methylphenidate increased risky helping by selectively altering risk- rather than social preferences. Atomoxetine influenced neither risk nor social preferences and did not affect risky helping. This suggests that methylphenidate-altered dopamine concentrations affect helping decisions that entail a risk to the helper.

## When Helping is Risky

Individuals differ in how much they help others. Such behavioral variation in helping is often considered a reflection of differences in social preferences – the value individuals assign to the welfare of another person (Fehr & Schmidt, 1999). However, helping others can put the helper at risk: When medical volunteers treat infectious patients, they risk to get the disease themselves. Or when a people attempt intervening in a fight, they risk getting injured themselves. As with social preferences, people also differ in the risks they are willing to take – the degree to which a decision maker tolerates uncertain outcomes (Frey, Pedroni, Mata, Rieskamp, & Hertwig, 2017) – and this might influence people’s willingness to help (e.g. Exley, 2016). Indeed, public debate about whether or not to help often revolves around the risks involved. For example, attempts to bring Ebola patients to hospitals in the United States were met with strong public concerns over infection risks (Yang, 2015).

How do people decide whether or not to help in situations where helping is risky? Is this determined by their social preferences, their risk preferences, or an interplay of both? And in the latter case, how are social and risk preferences integrated into a decision to help? Because social and risk preferences have been mostly studied in isolation, the precise form and function of these two preferences in determining whether people help in risky situations remains poorly understood. Here we examine how social and risk preferences are psychologically and neurobiologically integrated when helping comes at a risk to oneself.

Evidence suggests that the brain represents the possible consequences of different decision options on a common subjective value scale by integrating and trading off different option features or conflicting internal motives, like risk aversion or social preferences (Gross et al., 2014; Levy & Glimcher, 2012; McNamee, Rangel, & O’Doherty, 2013). Specifically, corticostriatal circuits encompassing the dorsolateral prefrontal cortex and the striatum associate with both risk-taking (Knoch et al., 2006; Mohr, Biele, & Heekeren, 2010) and social preferences (Baumgartner, Knoch, Hotz, Eisenegger, & Fehr, 2011; Christov-Moore, Sugiyama, Grigaityte, & Iacoboni, 2016; Crockett, Siegel, Kurth-Nelson, Dayan, & Dolan, 2017; Gross, Emmerling, Vostroknutov, & Sack, 2018). This corticostriatal circuitry is targeted by the neurotransmitter dopamine, which independently modulates both risk-taking (Fiorillo, Tobler, & Schultz, 2003; Schultz, Carelli, & Wigthman, 2015) and social preferences (Crockett et al., 2015; Sáez, Zhu, Set, Kayser, & Hsu, 2015; Soutschek et al., 2017, see Crockett & Fehr, 2014 for a review). For example, studies in rats and monkeys have shown that subcortical dopamine release is associated with risk-taking (Fiorillo et al., 2003; Simon et al., 2011) and that dopamine firing rates reflect the curvature of the utility function – the mathematical representation of subjective value that indicates risk attitudes (Schultz et al., 2015).

In humans, risk-taking increases following the administration of the dopamine precursor levodopa (Kobayashi, Asano, Matsuda, & Ugawa, 2019; Rigoli et al., 2016; Rutledge, Skandali, Dayan, & Dolan, 2015) and the D2/D3 agonist pramipexole (Campbell-Meikeljohn et al., 2011; Riba et al., 2008). Although there is less data on dopaminergic modulation of social preferences, levodopa has been shown to reduce generosity in dictator games (Pedroni, Eisenegger, Hartmann, Fischbacher, & Knoch, 2014) and hyper-altruism in a harm aversion task (Crockett et al., 2015), while tolcapone – a drug that enhances dopamine transmission in the prefrontal cortex – increases inequity aversion (Sáez et al., 2015).

Combined, these studies implicate a common neural circuitry for risk and social preferences that is sensitive to dopamine manipulations and may be engaged in risky helping decisions. Here we examined this possibility by manipulating dopaminergic neurotransmission using methylphenidate and atomoxetine. By blocking the dopamine and norepinephrine transporters, methylphenidate increases dopamine concentration in the striatum (Kim et al., 2009; Smith, Beveridge, & Porrino, 2006; Volkow et al., 2012), and both dopamine and norepinephrine concentration in the ventromedial prefrontal cortex and dorsolateral prefrontal cortex (Arnsten & Li, 2005). In line with findings on the dopaminergic modulation of risk preferences, studies have shown that methylphenidate can increase risk taking in rats (Yates et al., 2020) and humans (Campbell-Meiklejohn et al., 2012; Mandali, Sethi, Cercignani, Harrison, & Voon, 2021). Similar to methylphenidate, atomoxetine increases synaptic levels of both norepinephrine and dopamine in the prefrontal cortex. In contrast to methylphenidate, however, atomoxetine does not affect striatal dopamine levels (Bymaster et al., 2002; Schulz et al., 2017), and there is little evidence for norepinephrine modulation of risk or social preferences in humans (Crockett et al., 2010; Crockett & Fehr, 2014). Studies in rats suggest that norepinephrine modulates risk taking only when combined with dopamine reuptake inhibition (Montes, Stopper, & Floresco, 2015). By comparing the effects of methylphenidate and atomoxetine on behavior, it is possible to probe the differential contributions of dopaminergic versus noradrenergic systems on risky helping.

Based on previous studies, we hypothesize that methylphenidate will causally alter risk preferences. What remains unknown is whether methylphenidate also affects social preferences and if and how methylphenidate impacts the integration of both risk and social preferences and, thereby, influences helping decisions that come with a risk to oneself. Meanwhile, due to scant evidence implicating norepinephrine in risk or social preferences in humans, we did not expect to observe effects of atomoxetine on risky helping behavior. We performed two studies to examine these possibilities. In both studies, we used incentivized behavioral tasks that confront the individual with a decision to help or refuse to help another person when helping is not only costly but also risky. By manipulating risk and social consequences independently within the same task, we measured social and risk preferences in isolation to study how these preferences are integrated when helping is risky. Study 1 tested the integration of social and risk preferences by observing participants’ behavioral decisions when choices are risky, have consequences for others, or carry both risks to oneself and consequences for others. Study 2 complemented behavioral observations of Study 1 with a double-blind, placebo-controlled neuropharmacological intervention using methylphenidate and atomoxetine to manipulate dopaminergic and noradrenergic neurotransmission during decision making.

## Methods

### The Risky Helping Task

**Figure 1:**
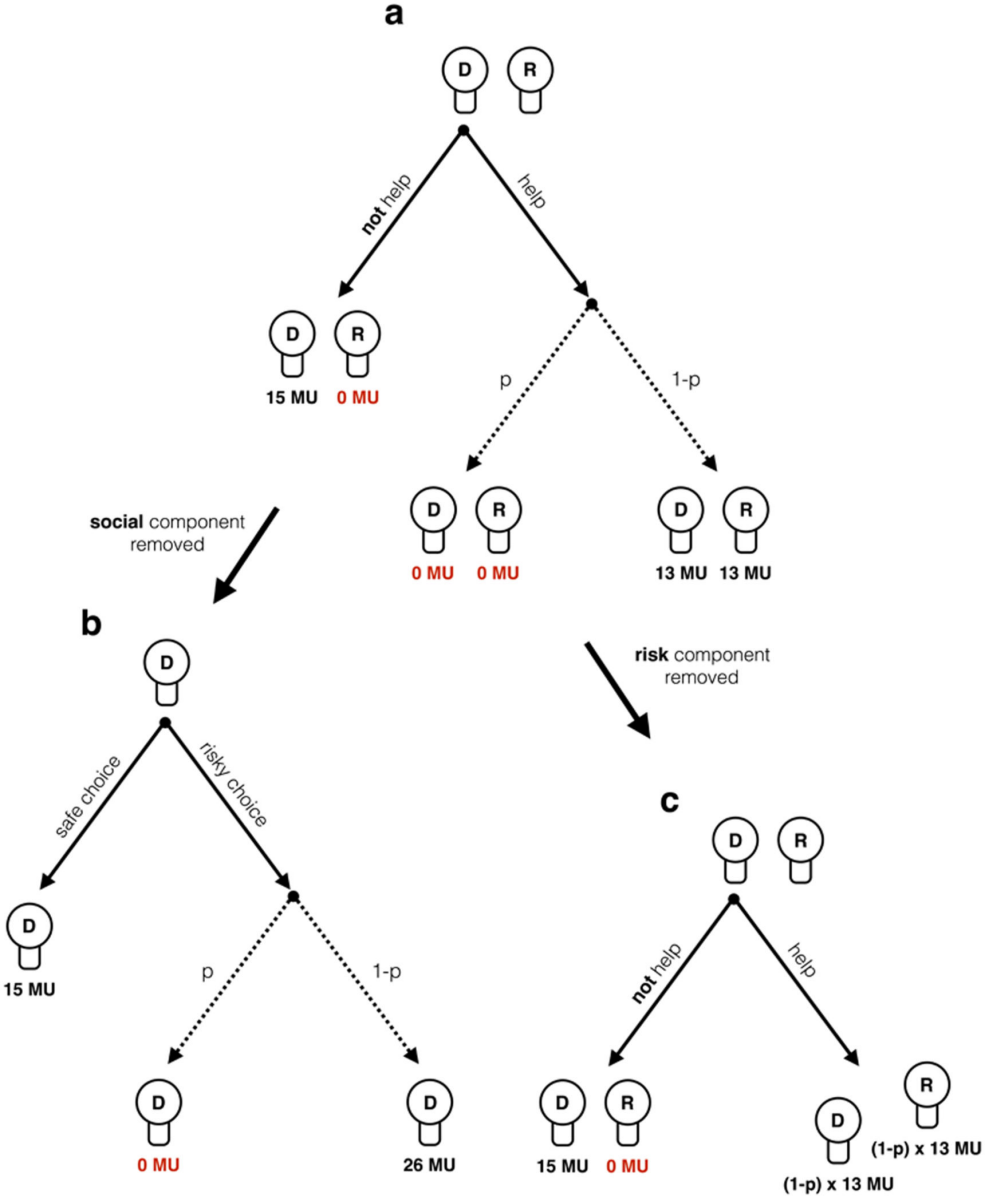
Risky helping, risk, and helping task. In the risky helping task **(a)** the decider (D) decides whether to help or not to help the receiver (R). In case the decider decides to help, helping is unsuccessful with probability *p* and both decider and receiver earn 0 monetary units (MU). With probability 1-*p* helping is successful, leading to an equal outcome of 13 MU for both. In the risk task **(b),** the decider decides between a safe option leading to a payoff of 15 MU and a risky option, leading to either a payoff of 0 MU with probability *p* or a payoff of 26 MU with probability 1-p. In the risk task, the receiver is not affected by the decider's decision. Thus, the social component of the risky helping task is removed. In the helping task **(c),** the decider decides whether to help or not to help the receiver. Across trials, helping leads to a sure outcome equal to the expected value of possible outcomes of the risky helping task. Thus, the risk component of the risky helping task is removed. In the actual task, the labels “help”, “not help”, “risky choice”, “safe choice” were replaced with neutral labels to avoid framing effects.

To investigate risky helping as a function of social and risk preferences, we designed a two-player incentivized “risky helping task” (Figure 1a). It involves a decider who repeatedly decides whether or not to help another participant, the receiver. The decision to refuse help leads to a sure outcome of 15 monetary units (MU) for the decider and zero MU for the receiver. The decision to help is risky: With a probability *p* helping is unsuccessful, in which case both decider and receiver earn zero MU. When helping is successful (with probability 1-*p*), both earn 13 MU. Note that helping always reduces inequality between decider and receiver to zero. Further, helping is costly to the decider: Even when successful, the decider incurs a cost of 2 MU. Hence, the risky helping task confronts the decision maker with a dilemma between avoiding risk to oneself and helping another person.

To measure the relative influence of the decider’s social and risk preferences, we derive two variants of the risky helping task, the “risk task” and the “helping task”. In the “risk task”, we remove the social component of the task to measure risk preferences in isolation. In this task, the receiver is not affected by the decisions of the decider. Instead, the decider chooses between a sure outcome and a lottery for each possible *p* that only affects her payoff (Figure 1b). In other words, the risk task confronts the decision maker with a dilemma between choosing a safe option or a gamble that can lead to a higher payoff (with probability 1-*p*) but also entails the risk to earn nothing for that trial (with probability *p*). The outcome for each decision option is the sum of money at stake in the risky helping task (i.e. 15 MU for the safe choice and 26 MU for the risky choice) to make sure that the safe choice would not simply dominate the risky choice in both risk and payoff for all *p.* In essence, the risk task measures risk preferences by reducing the risky helping task to a set of paired lottery choices that have consequences only for the decider but not for the other person.

In the “helping task” the decider chooses between the option to help or not help. Compared to the risky helping task, we remove the risk component to measure social preferences in isolation (Figure 1c). To achieve this, the helping outcome of the risky helping task is replaced with the expected value for each *p* (i.e. *p* × 0 + (1-*p*) × 13), as illustrated in Table 1. For example, in one trial of the risky helping task the decision maker faces the following choice:

(A) 15 MU for you, 0 MU for the other person
(B) with *p* = 0.5: 0 MU for you, 0 MU for the other person;
  with 1-*p* = 0.5: 13 MU for you, 13 MU for the other person

The equivalent trial in the helping task is:

(A) 15 MU for you, 0 MU for the other person
(B) 6.5 MU for you, 6.5 MU for the other person

Thus, the decider chooses between two non-risky outcomes in the helping task: One option maximizes own payoff but also leads to inequality, while the other option requires to sacrifice own MU to benefit the receiver and eliminate inequality (similar to a mini dictator game with different efficiency gains). In other words, the dilemma for the decision maker reduces to making a choice between a selfish option that leads to zero MU for the receiver and a pro-social option that benefits the receiver at a cost to the decider.

### Experimental Implementation

In the experiments, neutral labels for helping and risk were used to avoid demand or framing effects and decisions were completely anonymous to avoid reputation effects. In both studies, each participant performed the tasks in front of a personal computer terminal. Comprehension checks were used to make sure that participants understood the instructions (see Supplementary Information for task instructions and decision interface).

To measure risk and social preferences and the extent of helping in a risky environment on the individual level, each decider engaged in all three separate tasks (risky helping task, risk task, helping task) in random order (within-subject design). Probability *p*, i.e. risk, was systematically varied between 0 and 1 across 21 randomly presented trials in the risk task and the risky helping task.

The decisions of the decider had real financial consequences for decider and receiver that were paid out in cash at the end of the experimental session (one round of each task was selected at random for payment; 1 MU was worth 20 Euro cents in Study 1 and 36 British pennies in Study 2). In Study 1, receivers were also confronted with every decision that the decider had to face in the risk, helping, and risky helping task, but instead had to guess how the decider would decide. Guessing correctly was financially incentivized (see Supplementary Information for results).

### Participants and Procedure

In Study 1, 292 participants (mean age = 22.3±3.7, 142 female) were recruited and split into 146 deciders and 146 receivers. Sample size (aimed at ~N=300) for this within-subjects design was based on earlier work assessing individual differences in social value orientation (van Lange, 1999), typically finding roughly 45% (40%) of the sample holding pro-social (pro-self) preferences. Based on this heuristic we expected a good spread of social preferences in our sample, with ~50 participants having strong and ~50 participants having weak social preferences. Data were not analyzed before the full data collection was finished.

Participants were free to withdraw from participation at any time. Only individuals who voluntarily entered the recruiting database were invited and informed consent was obtained from all participants by electronic acceptance of an invitation to attend an experimental session. The experiment was conducted following the peer-approved procedures established by the Center for Research in Experimental Economics and Political Decision Making (CREED) at the University of Amsterdam, and was fully incentivized. The experiment was performed in a large lab with 30 separated cubicles. This allowed participants to see that other people took part in the experiment while not being able to identify with whom they were paired with or how other participants decided during the experiment. Following standard laboratory protocols, we did not use deception and invited participants from a pool of participants that were aware of this no-deception policy.

In Study 2, a sample of 154 participants (mean age = 23.7±3.9, 77 female) was recruited in the role of deciders, with only one participant taking part per experimental session (we aimed for 150 participants and analyzed data only upon completion of full data collection). This sample size equals the one used in Study 1 and follows our earlier work on drug administration and behavioral decision-making using ~50 participants per treatment able to detect small to medium effect sizes (Baas, Boot, van Gaal, De Dreu, & Cools, 2020; Crockett et al., 2015). Like in Study 1, data were not analyzed before the full data collection was finished.

Participants were free to withdraw from participation at any time. Participants completed a pre-screening questionnaire to select only those who had no history of drug consumption, a limited alcohol and caffeine uptake, no clinically relevant depression scores, and who were generally in a healthy condition. Participants underwent another screening via telephone the day before the study, to make sure they had, for example, not consumed any alcohol or other medication. The experiment received ethical approval from the University of Oxford’s Medical Sciences Interdivisional Research Ethics Committee. Because dopamine function can vary throughout the day (Hood et al., 2010), each experimental session took place in the same place at roughly the same time of the day (starting around 9 AM). Before substance administration and after pre-administration checks, female participants took a test to confirm they were not pregnant and we measured height, weight, and blood pressure of all participants. Supervised by a medical doctor throughout the study, participants either received 30mg of methylphenidate, 60mg of atomoxetine, or placebo, contained in an identical blue capsule. In line with ethical requirements, participants were informed about the three substances they might be taking, but neither them nor the experimenters nor the medical doctors knew which substance was administered to a participant. To make sure that testing coincided with peak absorption rate, the experiment started 90 minutes after drug administration. Participants then took part in the risk task, the helping task, and the risky helping task as part of a larger test battery. Participants also completed a 15-item visual analogue mood scale at two time-points of the experiment. Namely, before drug administration and after the experiment was finished. For each item, participants had to place a mark along a straight line with two opposing adjectives on either side (e.g. ‘muzzy vs. clearheaded’, ‘happy vs. sad’, ‘tense vs. relaxed’), which resulted in a continuous measure between 0 and 1 for each item. Hence, we measured subjective mood states before and after the drug administration (which also allows us to compute mood changes).

### Analyses

In each task, deciders made the binary decision to help or not (helping task and risky helping task), or to take the risky or the safe choice (risk task) across trials that differed in risk (risk / risky helping task) or helping consequences (helping task). In each trial we, hence, observed a binary decision. The binary decisions within each task revealed when a participant would take the risky/social option and when they would switch to the certain/selfish option. We used these switching points as a measure for risky helping, social preferences, and risk preferences.

For example, if a participant decided to choose the lottery for a risk *p* = [0, 0.05, 0.10, 0.15, 0.20, 0.25] and switched to the sure outcome for *p* = [0.30, ..., 1] in the risk task, the participant was assigned the value 0.30 as their measure of risk preference. Note that this implies that a switching point of 0.45 or lower indicates risk aversion (or risk neutrality), since the decision maker switched to the safe option before the expected value of the lottery decreased below the fixed payoff of the safe option. A switching point of 0.5 and higher indicates risk seeking, since the decision maker accepted lotteries for which the expected value of the lottery was lower than the fixed payoff of the safe option.

Between 14% and 18% of our participants had multiple switching-points, thereby violating monotonicity. This is not unexpected, since trials were randomly and sequentially presented within each task. Accordingly, we interpret violations of monotonicity as noise and averaged the switching points in these cases. Thus, a decision pattern like [**B**, **B**, **B**, A, **B**, A, A, …, A] for risk-levels of [0, 0.05, 0,10, 0.15, 0.20, 0.25, 0.30, ... 1] led to a switching point of 0.20. Excluding participants who violated monotonicity from the analysis did not alter the reported conclusions below (see Supplementary Information).

To analyze switching points, we used Tobit regressions. Tobit regressions deviate from a linear regression model in that they account for the (left) censoring in the data as they assume a latent variable y* that linearly depends on the predictors (y is equal to y* if y > 0, but deviates in the lower bound). For y = y*, we used the likelihood function of a *t*-distribution t(y|bX, σ^2^, df). For y ≠ y*, the likelihood function is based on the normal cumulative distribution and hence models the probability that y* will take a value less than or equal to y, given the observed predictors. This approach allows us to derive unbiased estimates for predictors and error variance in the presence of heteroscedasticity.

## Results

The Supplementary Information reports the regression models and results, alongside additional models that include control variables and robustness checks. Here we summarize the main findings from our two experiments.

### Study 1 Results

**Figure 2:**
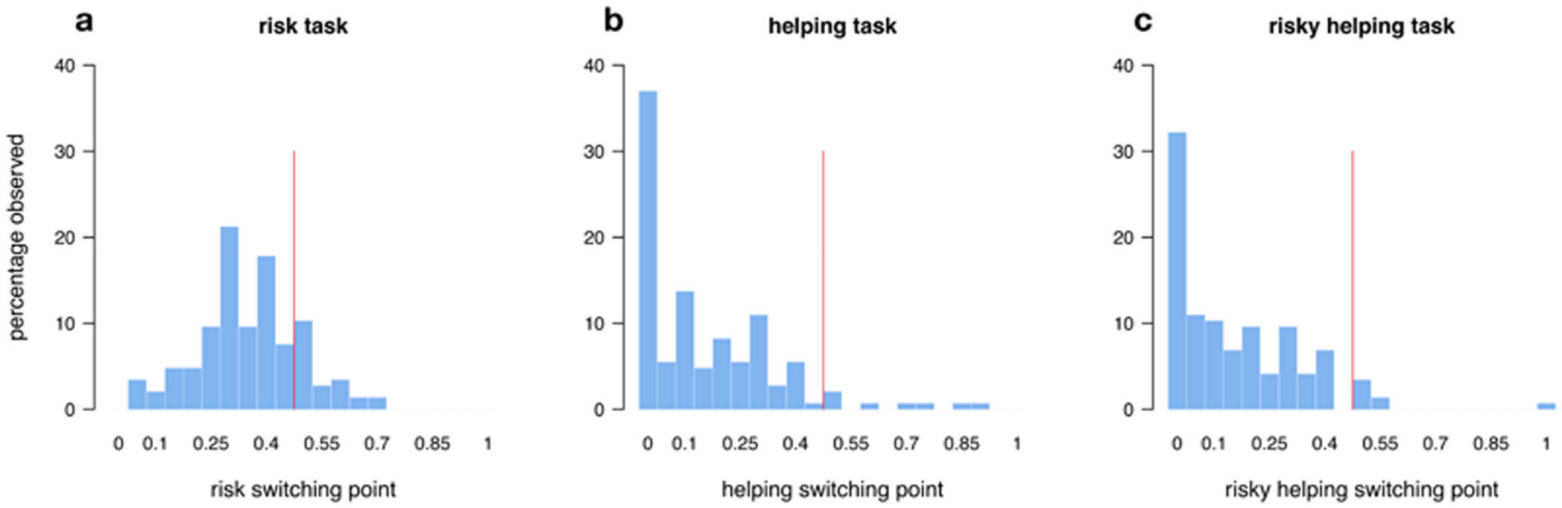
Switching points. Distribution of switching points across the risk **(a),** helping **(b),** and risky helping task **(c).** Red line indicates the border of risk aversion to risk seeking (risk task) and social efficiency to inefficiency (helping and risky helping task). Social efficiency refers to making helping/risky helping decision only when the combined (expected) payoff for decider and receiver exceeds the sure payoff of not helping. Having a switching point above the efficiency point means that participants chose to help even when the combined (expected) payoff for decider and receiver was lower than the sure payoff of 15 MU for not helping (i.e. 15 MU for the decider and 0 MU for the receiver).

In line with previous literature (Holt & Laury, 2002), participants were predominantly risk-averse in the risk task: They switched to the risky option only for gambles for which the expected value of the risky option exceeded the value of the safe option (Figure 2a). In the helping task, a majority of participants revealed social preferences. Most participants gave up some of their own resources to help the other person at least once (Figure 2b). In the risky helping task, participants who engaged in risky helping did so up to a risk of *p* = 0.15 on average, and then switched to not helping (Figure 2c). When participants engaged in risky helping, the gain to social welfare (i.e., the combined expected earnings of decider and receiver) significantly exceeded their own monetary sacrifice, showing that risky helping was not unconditional (reduction in earnings for deciders vs. expected value of decider and receiver; Wilcoxon Signed Rank test, p < 0.001, d = 0.66). On average, deciders sacrificed 1 MU to achieve an expected welfare increase of 1.8 MU.

To test if risky helping decisions can be modelled as a function of risk and social preferences, we analyzed if and how switching points in the risk and helping task predicted risky helping decisions. We found, first of all, that risk preferences derived from the risk task and social preferences derived from the helping task were linearly independent of each other (Spearman r = -0.02, p = 0.787). Thus, an individual’s social preference could not be inferred from knowing their risk preference and vice versa, pointing to a behavioral dissociation of risk and social preferences.

Yet, both risk and social preferences independently predicted risky helping decisions. We first calculated the change in switching points between helping and risky helping task on the individual level to see if this change was accounted for by risk preferences. The change in switching points between helping and risky helping was significantly correlated with the switching point in the risk task (Spearman r = 0.25, p = 0.002). The more risk-tolerant participants were (as measured in the risk task), the more they helped under risk (compared to no risk). Conversely, a switching point change between the risk task and the risky helping task was significantly correlated with the extent of helping in the helping task (Spearman r = 0.57, p < 0.001). In other words, stronger social preferences (as measured in the helping task) were associated with an increased willingness to help under risk (as measured in the risky helping task).

**Figure 3:**
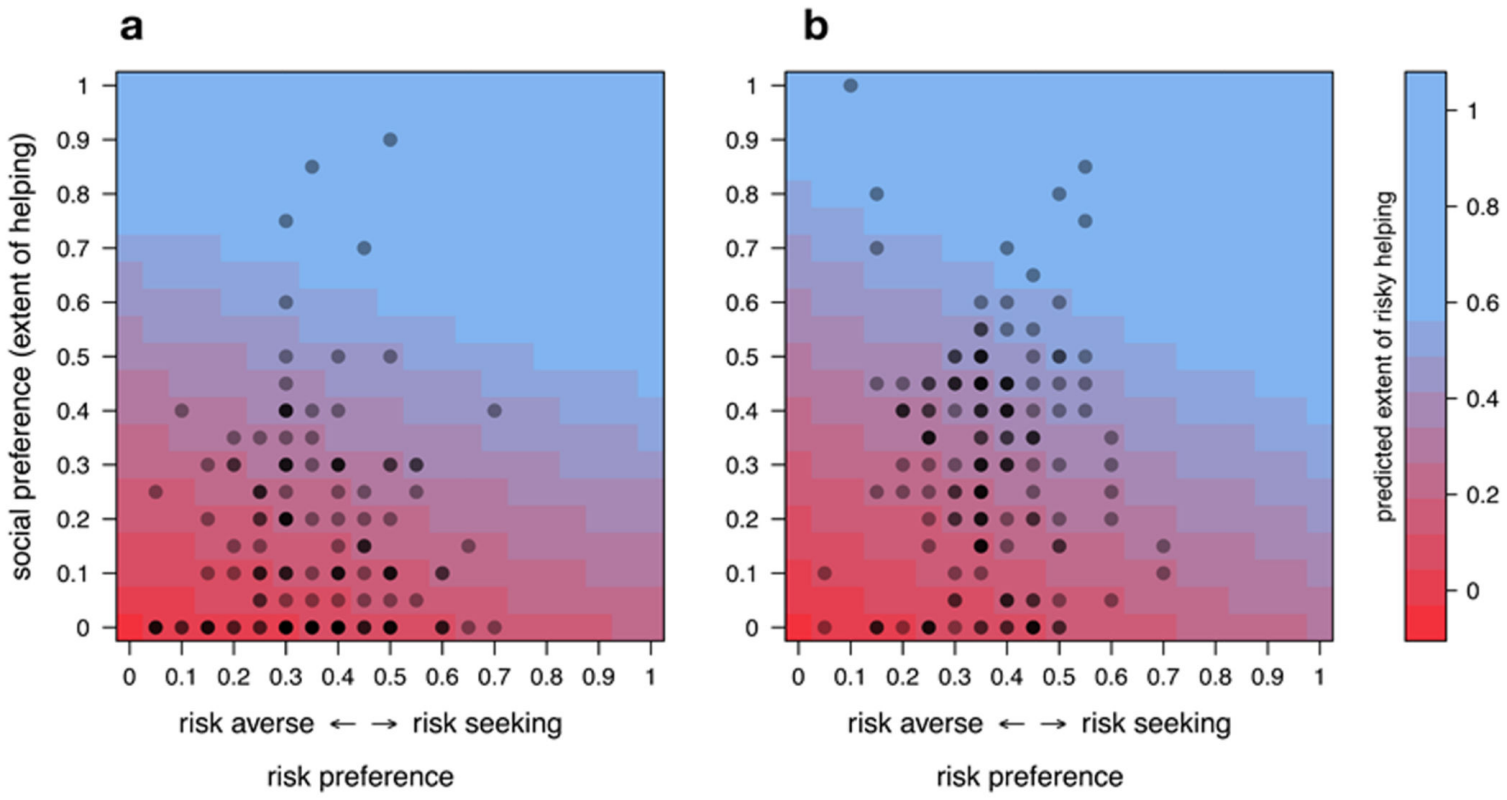
Predicting risky helping. Plot showing the predicted extent of risky helping (color surface) based on a linear combination of risk preference and social preference in Study 1 **(a)** and Study 2 **(b).** Dots show the observed risk (x-axis) and social preference (y-axis) of deciders. Darker dots indicate that multiple deciders had the same risk and social preference profile.

Another approach to test the extent to which risky helping can be predicted by risk preference, social preference, or their combination, is to use regression models. As illustrated in Figure 3a, risky helping decisions were a function of both social preferences and risk preferences (Tobit regression: risk preference estimate = 0.26, se = 0.09, p = 0.005, social preference estimate = 0.83, se = 0.07, p < 0.001). Comparing the relative weight of social and risk preferences also revealed that, while both variables were independently associated with risky helping, social preferences were a stronger predictor of risky helping than risk preferences (post-hoc test, H1: social preference estimate - risk preference estimate ≠ 0, estimate = 0.57, se = 0.12, p < 0.001). In other words, helping under risk emerged only with moderate to high social preferences combined with low to moderate risk aversion. Taken together, Study 1 provides evidence that risk and social preferences are behaviorally independent of each other, yet systematically integrated in situations where helping is risky.

### Study 2 Results

As in Study 1, individuals’ risk and social preferences were uncorrelated (Spearman r = 0.03, p = 0.800). Further replicating the results of Study 1, risk preferences were correlated with changes in helping between helping versus risky helping task (Spearman r = 0.24, p = 0.003). Participants who were more risk taking were also more likely to help when it was risky compared to when it was not. When participants were more risk averse, in contrast, they were less likely to help when it was risky. Within-individual changes in switching points between risk and risky helping task were also positively correlated with social preferences (Spearman r = 0.61, p < 0.001). Hence, participants with stronger social preferences accepted more risk when it could benefit another person (and the other way around).

As shown in Figure 3b, risky helping decisions could again be modelled as a linear combination of social preferences and risk preferences (Tobit regression: risk preference estimate = 0.31, se = 0.09, p = 0.001, social preference estimate = 0.73, se = 0.05, p < 0.001). This means that a person’s social preferences did not allow accurate predictions of risky helping without also factoring in this person’s risk preferences and vice versa. And in line with Study 1, we found that that social preferences were a stronger predictor of risky helping than risk preferences (post-hoc test, H1: social preference estimate - risk preference estimate ≠ 0, estimate = 0.42, se = 0.11, p < 0.001).

**Figure 4:**
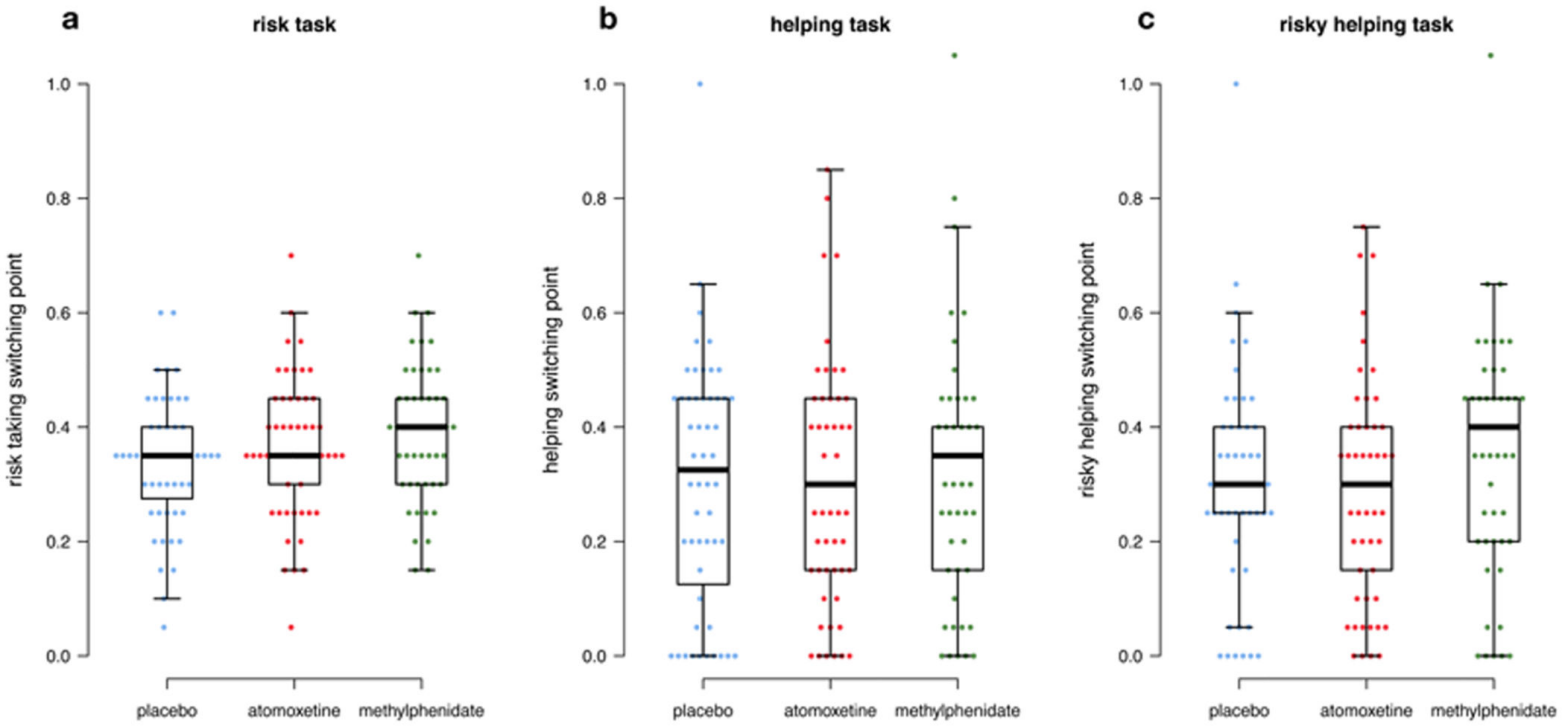
Preference changes across drug conditions. Distribution of switching points in the risk **(a),** helping **(b),** and risky helping task **(c).** Points show individual data points (blue = placebo, red = atomoxetine, green = methylphenidate).

Methylphenidate causally affected risk preferences (Figure 4a). Participants under methylphenidate accepted significantly more risk than participants under placebo (Tobit regression, methylphenidate vs. placebo estimate = 0.05, se = 0.02, p = 0.018). While 90% of the participants under placebo were classified as risk-averse (similar to Study 1), this dropped to 78% under methylphenidate. Risk-taking did not differ between placebo and atomoxetine (Tobit regression, atomoxetine vs. placebo estimate = 0.02, se = 0.02, p = 0.295). We also found no statistically significant difference in risk-taking between atomoxetine and methylphenidate (Tobit regression, atomoxetine vs. methylphenidate estimate = 0.03, se = 0.02, p = 0.185).

There was no evidence that methylphenidate or atomoxetine affected social preferences (Figure 4b, Tobit regression: methylphenidate vs. placebo estimate = 0.02, se = 0.05, p = 0.728; atomoxetine vs. placebo estimate = 0.00, se = 0.05, p = 0.947; methylphenidate vs. atomoxetine estimate = 0.02, se = 0.05, p = 0.664). However, due to their higher willingness to take risks, participants under methylphenidate helped more often in the risky helping task compared to, both, atomoxetine and placebo (Figure 4c, Tobit regression: atomoxetine vs. methylphenidate effect estimate = 0.08, se = 0.04, p = 0.030; methylphenidate vs. placebo estimate = 0.07, se = 0.04, p = 0.047). In contrast, atomoxetine did not increase risky helping compared to placebo (Tobit regression: atomoxetine vs. placebo estimate = -0.01, se = 0.04, p = 0.787). Consequently, participants under methylphenidate sacrificed more resources in the risky helping task, which resulted in higher overall welfare compared to placebo and atomoxetine. Under methylphenidate, 22.4% of participants helped even beyond the social efficiency point under risk, compared to 11.5% and 13.2% under placebo and atomoxetine, respectively.

The effects of methylphenidate on risk attitudes and risky helping may be driven by interindividual differences in drug-absorption rates, drug interactions with age or gender, or effects of drugs on mood. To examine these alternative explanations, we controlled for age and gender in the reported regressions and computed additional regressions where we controlled for body-mass index and regular medication (e.g. contraceptive pill) that both may influence absorption rates of the drug agent. We also controlled for mood and mood changes that have been shown to influence risk-taking and social decision making. Across these control analyses, drug treatment remained a robust predictor of risk and risky helping decisions (Table S6-S8). Further, when asked to guess what treatment they received, 38% of the participants guessed correctly (which is consistent with random guessing, i.e. chance level of 33%, chi-square test, χ^2^(1) = 144, p = 0.230). Repeating the reported analyses only with participants that incorrectly guessed the treatment did not change the above conclusions (Table S6-S8).

Another possibility is that drug treatment makes decision makers more erratic and as such increases choice inconsistency. This may have led to a spurious increase or decrease in switching points. Choice consistency in our tasks can be measured by looking at the number of switching points. A perfectly consistent decision maker has one unique switching point in each task, while multiple switching points indicate intransitive choice. In the risk task, 76% of the participants had a unique switching point (compared to 86% in Study 1). In the helping task, 73% of the participants had a unique switching point (compared to 82% in Study 1). Lastly, in the risky helping task, 77% of the participants had a unique switching point (compared to 83% in Study 1). While participants were slightly less consistent than in Study 1, we found no statistical evidence that the drug treatments made decisions noisier and more inconsistent in the risk task (Tobit regression, methylphenidate vs. placebo estimate = 0.50, se = 0.98, p = 0.609, atomoxetine vs. placebo estimate = 0.89, se = 0.92, p = 0.337), helping task (Tobit regression, methylphenidate vs. placebo estimate = -1.55, se = 1.06, p = 0.141, atomoxetine vs. placebo estimate = 0.74, se = 0.72, p = 0.306), or risky helping task (Tobit regression, methylphenidate vs. placebo estimate = -0.24, se = 1.43, p = 0.862, atomoxetine vs. placebo estimate = 1.64, se = 1.28, p = 0.201). Reported results also remained robust when controlling for choice consistency in the regression model (Table S6-S8).

## Discussion

Helping can have unforeseen negative consequences for the helper that render pro-social behavior not only costly but also risky. Here we identify individual differences in social preferences that predict willingness to help, and risk preferences that predict willingness to take risks, and we show that people systematically integrate their risk preferences with their social preferences when helping is risky. Hence, we demonstrate that one cannot reliably predict decision-making in risky helping situations based on a person’s risk or social preferences alone. Instead, someone who refuses to help under risk could be driven by a lack of social preferences or by risk aversion (or a combination of both). This means that refusals to help others can be rooted in risk-aversion rather than selfishness. In contrast, our data on receivers also revealed that they often misattributed failures to help to a lack of social concern rather than a lack of risk tolerance (see Supplementary Information for further details on these results). Thus, interventions aimed at promoting helping under risk might need to be adjusted towards reducing risk-aversion, for example by emphasizing when fears of risk are unfounded, rather than at increasing people’s moral obligation to care for the welfare of others independent of risks.

As participant’s risk and social preferences were uncorrelated, our results suggest that both preferences are, to some degree, independently processed and then systematically integrated when social concerns need to be traded off against risk concerns. In line with this, methylphenidate led to more helping under risk because the substance selectively altered risk preferences but not social preferences. Meanwhile, atomoxetine changed neither risk nor social preferences and, as a result, also did not affect risky helping. Atomoxetine increases both norepinephrine and dopamine levels in the prefrontal cortex. Methylphenidate also inhibits norepinephrine reuptake but, in contrast to atomoxetine, also enhances dopamine neurotransmission in subcortical brain structures like the striatum (Bymaster et al., 2002; Schulz et al., 2017). Our results fit the possibility that risk preferences are modulated by striatal rather than prefrontal dopamine (Fiorillo et al., 2003; Onge, Chiu, & Floresco, 2010). Furthermore, because both drugs increase norepinephrine levels, yet only methylphenidate modulated risk preferences, results also suggest that if norepinephrine modulates risk preferences, it may do so especially – or only – when combined with striatal dopamine reuptake inhibition (Montes et al., 2015). Finally, our results suggest that subcortical dopamine transmission appears not to modulate social preferences. Hence, the effects of dopaminergic manipulations on social preferences found in previous research might be partly attributable to altered risk preferences.

From a practical perspective, both methylphenidate (sold under the trade name Ritalin) and atomoxetine (sold under the trade name Strattera) are prescription drugs used to treat attention deficit hyperactivity disorder (ADHD) and are regularly used off-label by people who aim to enhance their cognitive performance (Maier, Ferris, & Winstock, 2018). As such, our results have implications for the ethics of, and policy for the use of psychostimulants. Indeed, the Global Drug Survey taken in 2015 and 2017, revealed that 3.2% and 6.6% of responders reported to use psychostimulants like methylphenidate for cognitive enhancement (Maier et al., 2018). Both in the professional ethical debate as well as in the general public, concerns about the medical safety and the fairness of such cognitive enhancements are discussed (Faber, Savulescu, & Douglas, 2016). However, our finding that methylphenidate alters helping behavior through increased risk-seeking demonstrates that substances aimed at changing cognitive functioning can also influence social behavior. Such “social” side-effects of cognitive enhancement (may they be deemed positive or negative) are currently unknown to both users and administrators and thus do not receive much attention in the societal debate about psychostimulant use (Faulmüller, Maslen, & de Sio, 2013).

Conclusions are constrained by some limitations of our study. Since our sample consisted of healthy volunteers only and because drugs like methylphenidate can have different effects in clinical vs. non-clinical populations (Moll, Heinrich, & Rothenberger, 2003), it is not possible to generalize our results to clinical populations who take these drugs for medical purposes. Second, our results suggest that behavioral differences in risky helping are driven by the dopamine-specific effects of methylphenidate but our conclusions about the possible role of norepinephrine are largely based on null findings. Future research could be focused on these issues, using larger samples and neuroimaging techniques that provide more precision with respect to observing neurobiological mechanisms. Third, individuals in our experiments knew the exact probabilities associated with different decision-outcomes. Oftentimes, however, probabilities are less well-defined thus creating ambiguity rather than risk. The Ellsberg paradox demonstrates that people are ambiguity averse; they prefer bets with known rather than unknown probability distributions of the outcome space. Vives & FeldmanHall (2018) showed that interindividual differences in ambiguity tolerance, but not risk attitudes, predicted pro-social choice and trust (also see FeldmanHall, Glimcher, Baker, & Phelps, 2016). Future work is needed to identify how the biobehavioral dissociation of risk and social preferences shown here generalizes to ambiguous situations.

## Conclusion

Decision makers can rarely be sure about how their decisions affect other people or their own welfare. Accordingly, social decisions often require an integration of social preferences and risk concerns that, theoretically, can have divergent effects on decisions and create a dilemma between helping others and avoiding risk to oneself. Our results highlight that risk preferences and social concerns are orthogonal, behaviorally dissociable preferences. A failure to help can reflect a lack of concerns for others or a personal aversion to risk – or both.

While previous research revealed that risk and social consequences are processed in overlapping neural circuitries, our drug manipulation suggests largely independent mechanisms. Methylphenidate selectively altered risk tolerance and left social preferences unchanged—it increased helping under risk but not helping without risk. Atomoxetine, on the other hand, had no effect on either risk or social preferences. These drug-induced changes in risk-taking and risky helping thus reveal a neurobiological dissociation of the processing of risk and social consequences in humans.

## Supplementary Material

Supplemental material

## Figures and Tables

**Table 1:** Decision consequences in the risk, helping and risky helping task depending on *p*. Expected value (EV), social welfare and earnings change (when the decider chooses to help, Option B) across risk levels (*p*) in the risk and helping/risky helping task. Each row represents one decision that the decider was faced with. In the risk task, the participant had to choose between a safe option (Option A) that always led to a return of 15 MU or a risky option (Option B), that led to a different expected value (EV) and therefore different expected earnings change when choosing Option B compared to Option A (last column) depending on *p.* In the helping/risky helping task, Option A refers to the non-helping (helping task) and safe (risky helping task) option that led to an outcome of 15 for the decider and 0 for the receiver. The expected value of the helping/risky-helping option (Option B) changed depending on *p*. The ‘(expected) welfare change’ column shows the change in expected total earnings (i.e. expected earnings of decider and receiver combined) when the decider would choose Option B as opposed to Option A. The ‘decider’s (expected) earnings change’ column shows the change in (expected) earnings for the decider when they would choose Option B as opposed to Option A.

		*Option A* (safe / non-helping option)		*Option B* (helping / risky-helping option)			
	*p*	EV decider	EV receiver	EV decider	EV receiver	(expected) welfare change of option B	decider's (expected) earnings change
risk task
	**1**	15	–	0	–	–	-15
	**0.9**	15	–	2.6	–	–	-12.4
	**0.8**	15	–	5.2	–	–	-9.8
	**0.7**	15	–	7.8	–	–	-7.2
	**0.6**	15	–	10.4	–	–	-4.6
	**0.5**	15	–	13	–	–	-2
	**0.4**	15	–	15.6	–	–	0.6
	**0.3**	15	–	18.2	–	–	3.2
	**0.2**	15	–	20.8	–	–	5.8
	**0.1**	15	–	23.4	–	–	8.4
	**0**	15	–	26	–	–	11
helping / risky helping task
	**1**	15	0	0	0	-15	-15
	**0.9**	15	0	1.3	1.3	-12.4	-13.7
	**0.8**	15	0	2.6	2.6	-9.8	-12.4
	**0.7**	15	0	3.9	3.9	-7.2	-11.1
	**0.6**	15	0	5.2	5.2	-4.6	-9.8
	**0.5**	15	0	6.5	6.5	-2	-8.5
	**0.4**	15	0	7.8	7.8	0.6	-7.2
	**0.3**	15	0	9.1	9.1	3.2	-5.9
	**0.2**	15	0	10.4	10.4	5.8	-4.6
	**0.1**	15	0	11.7	11.7	8.4	-3.3
	**0**	15	0	13	13	11	-2

## Statement of Relevance

People help others at sometimes substantial costs to themselves. What has been largely overlooked is that helping can not only be costly, but also be risky. When, for example, medical volunteers treat patients with infectious diseases, they may become infected themselves. Volunteers who try to rescue shipwrecked refugees risk injury or drowning. Here we identify individual differences in social preferences (predicting willingness to help) and risk preferences (predicting willingness to take risks) and examine how these distinct preferences alone and in combination predict decision-making when helping comes at a risk to the helper. We find that even people with high social preferences are more likely to help in risky situations when their risk tolerance is sufficiently high. Our findings both advance neurobiological theory and have practical implications by further showing that the drug methylphenidate increases risky helping because it alters risk rather than social preferences.
