# Supplementary material for: When Helping is Risky: The Behavioral and Neurobiological Tradeoff of Social and Risk Preferences
Source: Psychol Sci. Author manuscript; Available in PMC 2023 Jan 24. (PMC7614101; doi:10.1177/09567976211015942)

Supplementary Information

When Helping is Risky:
The Behavioral and Neurobiological Tradeoff of Social and Risk Preferences

Task implementation and instructions. Study 1 was conducted in a lab with 30 cubicles that are separated by divider walls. Participants were invited in groups of 14 to 22 participants and were randomly allocated to be in the role of a 'decider' or 'receiver'. Instructions were presented on the computer screen. Figure S1 shows the instructions for the risky helping task. Deciders were told that, in each trial, they had to choose between an 'Option A' and an 'Option B' and that, if they choose option A, they would always receive 15 MU and that their receiver would receive 0 MU. For option B, they were told that the 'outcome is not certain'. It could happen that they either receive 13 MU and their receiver would receive 13 MU or that both of them would receive 0 MU and that the outcome would depend on a chance level that changed across trials. Receivers in Study 1 received similar instructions, except that it was explained to them that their task was to guess what their decider would choose in each trial. Deciders were not told that the task of receivers was to guess what they would choose. This was done because the guesses from receivers were not payoff-relevant for deciders and to avoid any 'image concerns' that could be triggered by thinking about what the receiver may think about how oneself would decide.

After the instructions, all participants had to answer a set of comprehension questions before they could begin the task (Figure S2). Figure S3 shows the layout of the decision screen for deciders (Figure S3a) and receivers (Figure S3b). To make a decision, the participant had to select the answer with their mouse and then press the 'submit' button with their mouse. There was no time limit for making decisions. Hence, we did not induce any time pressure. After submitting a decision,

the decision options and submit button faded out (i.e. slowly became invisible, which took 1 second) and the next trial started by fading in two new decisions options (i.e. became visible, which took 1 second). The reminder explanation on the top of the screen remained visible throughout the whole task.

In the risk task, it was explained to participants that 'option A' always leads to a sure outcome for the decider, whereas 'option B' is a risky option that could also lead to 0 MU (Figure S4). After answering a set of comprehension questions (Figure S5), participants could proceed to the task. Figure S6 shows the screen-layout for two exemplary trials from the perspective of a decider and a receiver. Finally, Figure S7 shows how the instructions of the helping task were presented on the screen, Figure S8 shows the comprehension check of the helping task, and Figure S9 shows the screen-layout for two exemplary trials from the perspective of a decider and a receiver in the helping task. In the instructions and during the task, we did not use terms like 'risk', 'helping', or 'losing' to avoid any framing effects. Instead we used the terms 'probability' and 'outcomes'. Tasks were performed in random order and participants only received instructions for the next task after they completed the previous task.

Instructions for Study 2 remained unchanged, except that (a) the payment was converted to pounds instead of euros and (b) we did not mention that participants were paired with another person 'in this room', since the task was performed individually instead of in a large behavioral lab, as Study 1. Individual testing was necessary due to medical and ethical requirements for the drug study. Further, we did not invite separate receivers for Study 2. Instead, deciders were randomly paired among each other. Hence, each decider acted as a receiver for a different decider. Yet, this was not disclosed in the instructions to the task to avoid any (indirect) reciprocity concerns. Everything else remained as in Study 1, like the layout of the decision screen or the input method.

A common concern in interactive studies performed in front of a computer is whether participants believe that their decisions actually influence the outcome of other participants. In Study 1, we selectively invited participants from a pool of participants that are specifically recruited to take part in interactive studies that involve no deception. Also, the lab in which the study was performed has a strict no-deception policy. Further, the experiment was performed in a large room with other participants, allowing to see that other participants are present (albeit, not allowing to deduce with whom you, as a participant, are paired with, or seeing decisions of other participants). We are therefore confident that participants found it credible that their decisions actually influenced the payoff of another person. In Study 2, we did not run the experiment in a large behavioral economics lab due to the constraints of the drug-administration protocol that required only one participant being measured at a time. However, instructions clearly specified that decisions would affect matched responders who participated in an earlier or later session and that payouts would be calculated accordingly.

Figure S1. *Risky helping task instructions.* Instructions for (a) deciders and (b) receivers in the risky helping task.

a

For this task, you have been randomly paired with another person in this room. You have been assigned the role as the **Decider**, the other person will be the Receiver. You will make a series of decisions that affect both you and the Receiver. The Receiver and you will remain mutually anonymous. That is, she/he will learn how "her/his" Decider decided, but not who you are.

There will be 21 rounds of this task. In each round, you have to choose between action A and action B. The action you choose affects the payoffs that you and the Receiver will get. Payoffs are measured in monetary units (**MUs**). Each MU is worth 0.2 Euros, which will be paid out in the end.

If you choose **action A**, the outcome is certain. You will get **15 MUs** and the Receiver will get **0 MUs**.

If you choose **action B**, the outcome is not certain. <u>Either</u>, you will get **13 MUs** and the Receiver will get **13 MUs**. <u>Or</u> both of you will get **0 MUs**. Which of these two outcomes is going to happen depends on a chance that changes in the rounds of the task. For example, in one round there might be an 80% chance that you and the Receiver get 13 MUs and a 20% chance that you and the Receiver get 0 MUs. In another round, the chance might be 50% to 50%. You will learn about these chances in each round before you have to decide between action A and action B.

After you have made your choice, the round is over. Only one round will count "for real." This round will be randomly selected after you have made all 21 choices. Each round has an equal chance of being selected, so you should make each decision as if it was the one that counts. In case you have chosen action B in the round that gets selected, the outcome will be determined with the given chances, and you and the Receiver will get the payment depending on the outcome.

I read and understood the instructions

b

For this task, you have been randomly paired with another person in this room. The other person has been assigned the role as the **Decider**.

The Decider will make a series of decisions. There will be 21 rounds of this task. In each round, she/he has to choose between action A and action B. The action the Decider chooses affects the payoff she/he will get. Payoffs are measured in monetary units (**MUs**). Each MU is worth 0.2 Euros, which will be paid out to the Decider at the end.

If the Decider choose **action A**, the outcome is certain. She/he will get **15 MUs**.

If the Decider choose **action B**, the outcome is not certain. <u>Either</u>, she/he will get **26 MUs** <u>or</u> she/he will get **0 MUs**. Which of these two outcomes is going to happen depends on a chance that changes in the rounds of the task. For example, in one round there might be an 80% chance that the decider gets 26 MUs and a 20% chance that the decider gets 0 MUs. In another round, the chance might be 50% to 50%. The decider will learn about these chances in each round before she/he has to decide between action A and action B.

After the Decider has made her/his choice, the round is over. Only one round will count "for real." This round will be randomly selected after the Decider has made all of her/his 21 choices. Each round has an equal chance of being selected, so she/he should make each decision as if it was the one that counts. In case the Decider has chosen action B in the round that gets selected, the outcome will be determined with the given chances, and she/he will get the payment depending on the outcome.

<u>Your task</u> is to guess what the Decider chooses in each round. If you guessed the decision of the Decider correctly in the round that gets selected, you will receive an extra payment of 5 MUs.

I read and understood the instructions

Figure S2. *Risky helping task comprehension check.* Comprehension questions that were asked before participants could proceed to the task (correct answers are highlighted).

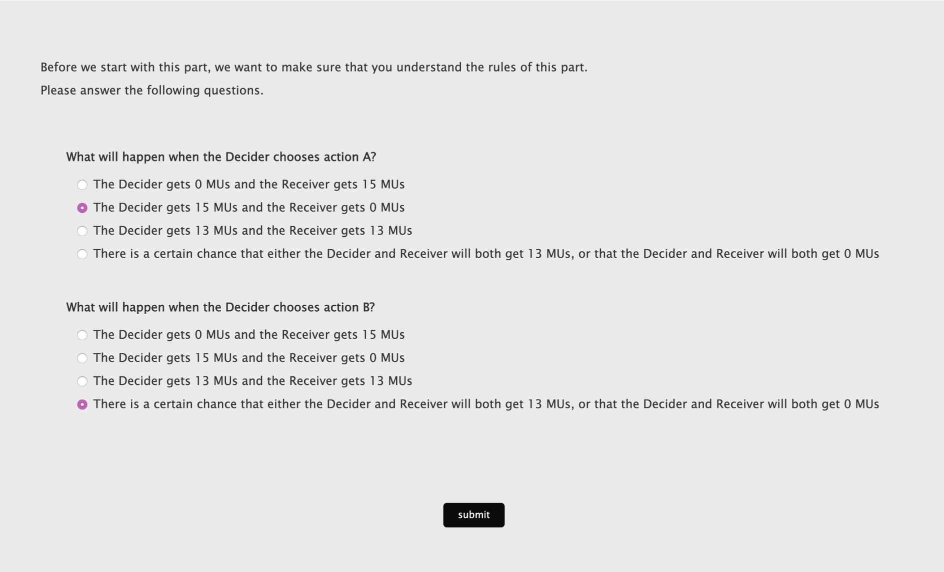

Figure S3. *Risky helping task.* Screenshots of two trials of the risky helping task from the perspective of (a) a decider and (b) a receiver (exemplary answers are highlighted for illustration).

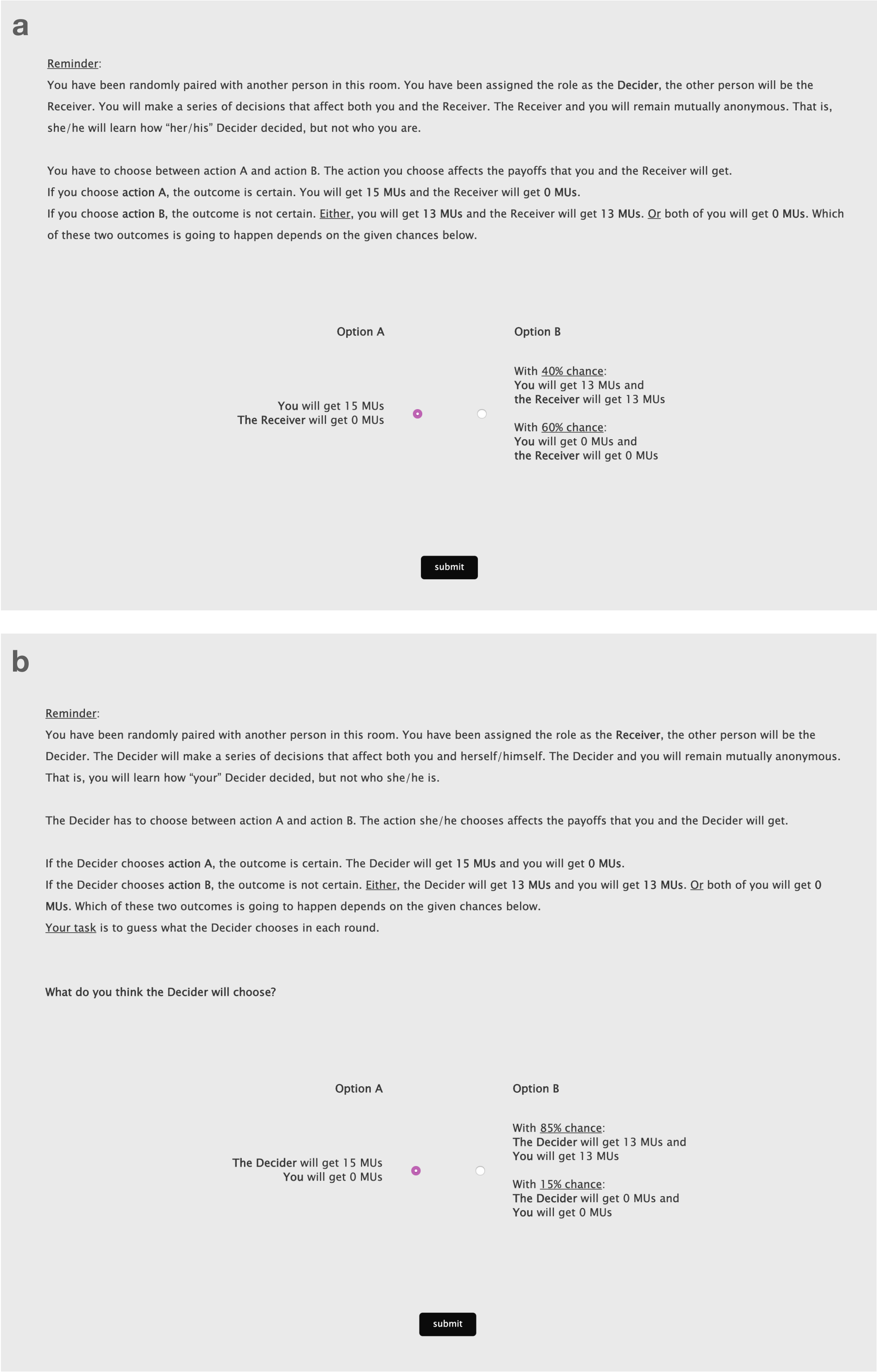

Figure S4. *Risk task instructions.* Instructions for (a) deciders and (b) receivers in the risk task.

a

In this task, you will make a series of decisions. There will be 21 rounds of this task. In each round, you have to choose between action A and action B. The action you choose affects the payoff you will get. Payoffs are measured in monetary units (**MUs**). Each MU is worth 0.2 Euros, which will be paid out in the end.

If you choose **action A**, the outcome is certain. You will get **15 MUs**.

If you choose **action B**, the outcome is not certain. Either, you will get **26 MUs** or you will get **0 MUs**. Which of these two outcomes is going to happen depends on a chance that changes in the rounds of the task. For example, in one round there might be an 80% chance that you get 26 MUs and a 20% chance that you get 0 MUs. In another round, the chance might be 50% to 50%. You will learn about these chances in each round before you have to decide between action A and action B.

After you have made your choice, the round is over. Only one round will count "for real." This round will be randomly selected after you have made all your 21 choices. Each round has an equal chance of being selected, so you should make each decision as if it was the one that counts. In case you have chosen action B in the round that gets selected, the outcome will be determined with the given chances, and you will get the payment depending on the outcome.

I read and understood the instructions

b

For this task, you have been randomly paired with another person in this room. The other person has been assigned the role as the **Decider**.

The Decider will make a series of decisions. There will be 21 rounds of this task. In each round, she/he has to choose between action A and action B. The action the Decider chooses affects the payoff she/he will get. Payoffs are measured in monetary units (**MUs**). Each MU is worth 0.2 Euros, which will be paid out to the Decider at the end.

If the Decider choose **action A**, the outcome is certain. She/he will get **15 MUs**.

If the Decider choose **action B**, the outcome is not certain. Either, she/he will get **26 MUs** or she/he will get **0 MUs**. Which of these two outcomes is going to happen depends on a chance that changes in the rounds of the task. For example, in one round there might be an 80% chance that the decider gets 26 MUs and a 20% chance that the decider gets 0 MUs. In another round, the chance might be 50% to 50%. The decider will learn about these chances in each round before she/he has to decide between action A and action B.

After the Decider has made her/his choice, the round is over. Only one round will count "for real." This round will be randomly selected after the Decider has made all of her/his 21 choices. Each round has an equal chance of being selected, so she/he should make each decision as if it was the one that counts. In case the Decider has chosen action B in the round that gets selected, the outcome will be determined with the given chances, and she/he will get the payment depending on the outcome.

Your task is to guess what the Decider chooses in each round. If you guessed the decision of the Decider correctly in the round that gets selected, you will receive an extra payment of 5 MUs.

I read and understood the instructions

Figure S5. *Risk task comprehension check.* Comprehension questions that were asked before participants could proceed to the task (correct answers are highlighted).

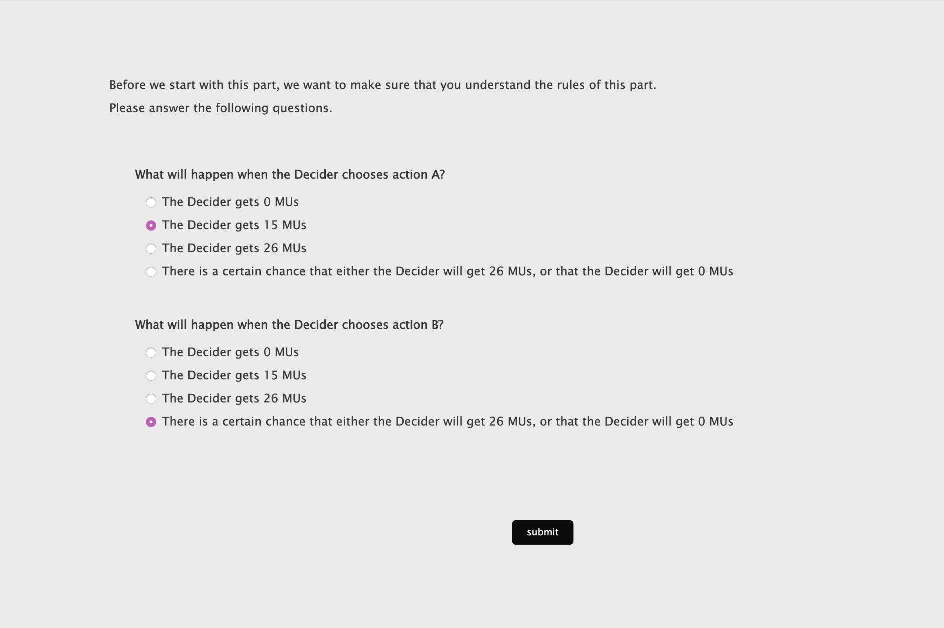

Figure S6. *Risk task.* Screenshots of two trials of the risk task from the perspective of (a) a decider and (b) a receiver (exemplary answers are highlighted for illustration).

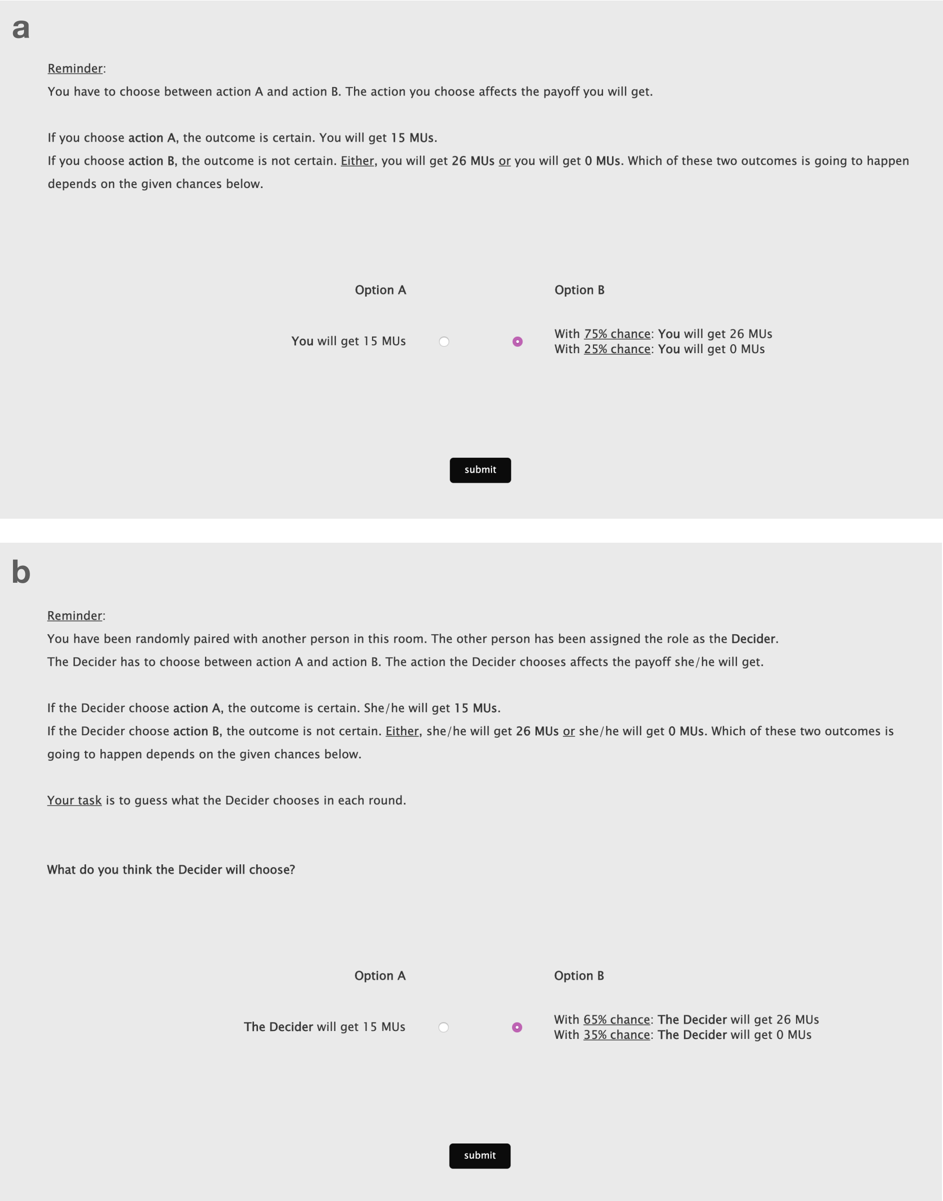

Figure S7. *Helping task instructions.* Instructions for (a) deciders and (b) receivers in the helping task.

a

For this task, you have been randomly paired with another person in this room. You have been assigned the role as the **Decider**, the other person will be the Receiver. You will make a series of decisions that affect both you and the Receiver. The Receiver and you will remain mutually anonymous. That is, she/he will learn how "her/his" Decider decided, but not who you are.

There will be 21 rounds of this task. In each round, you have to choose between action A and action B. The action you choose affects the payoffs that you and the Receiver will get. Payoffs are measured in monetary units (**MUs**). Each MU is worth 0.2 Euros, which will be paid out in the end.

If you choose **action A**, the outcome is always the same. You will get 15 **MUs** and the Receiver will get 0 **MUs**.

For **action B**, the outcome is different in each round. You will learn about the outcome of action B before you have to decide between action A and action B.

After you have made your choice, the round is over. Only one round will count "for real." This round will be randomly selected after you have made all 21 choices. Each round has an equal chance of being selected, so you should make each decision as if it was the one that counts. In the end, you and the Receiver will get the payment depending on your decision.

I read and understood the instructions

b

For this task, you have been randomly paired with another person in this room. You have been assigned the role as the **Receiver**, the other person will be the Decider. The Decider will make a series of decisions that affect both you and herself/himself. The Decider and you will remain mutually anonymous. That is, you will learn how "your" Decider decided, but not who she/he is.

There will be 21 rounds of this task. In each round, the Decider has to choose between action A and action B. The action she/he chooses affects the payoffs that you and the Decider will get. Payoffs are measured in monetary units (**MUs**). Each MU is worth 0.2 Euros, which will be paid out in the end.

If the Decider chooses **action A**, the outcome is always the same. The Decider will get 15 **MUs** and you will get 0 **MUs**.

For **action B**, the outcome is different in each round. The Decider will learn about the outcome of action B before she/he has to decide between action A and action B.

After the Decider has made her/his choice, the round is over. Only one round will count "for real." This round will be randomly selected after the Decider has made all 21 choices. Each round has an equal chance of being selected, so the Decider should make each decision as if it was the one that counts. In the end, you and the Decider will get the payment depending on her/his decision.

Your task is to guess what the Decider chooses in each round. If you guessed the decision of the Decider correctly in the round that gets selected, you will receive an extra payment of 5 MUs.

I read and understood the instructions

Figure S8. *Helping task comprehension check.* Comprehension questions that were asked before participants could proceed to the task (correct answers are highlighted).

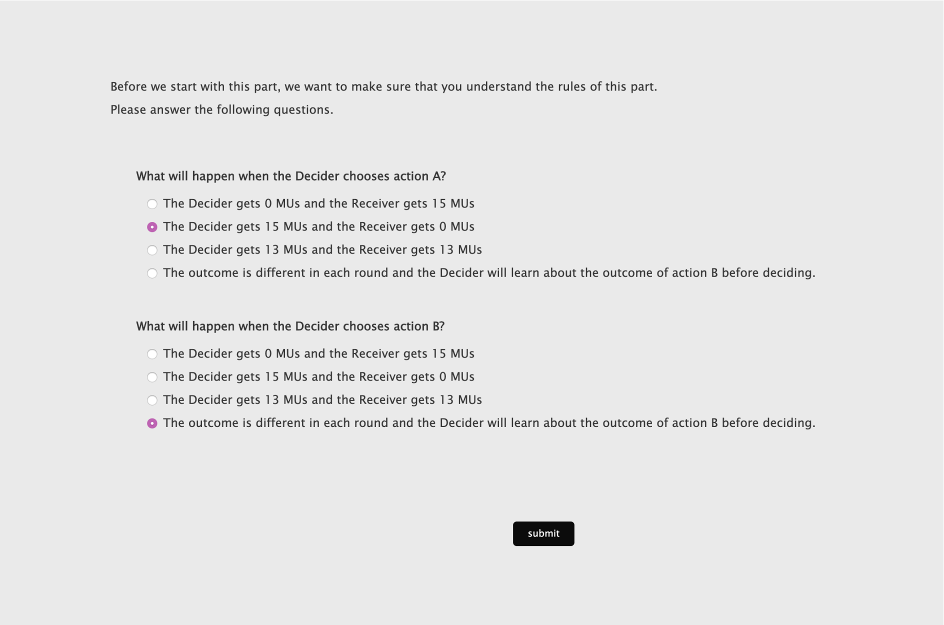

Figure S9. *Helping task.* Screenshots of two trials of the helping task from the perspective of (a) a decider and (b) a receiver (exemplary answers are highlighted for illustration).

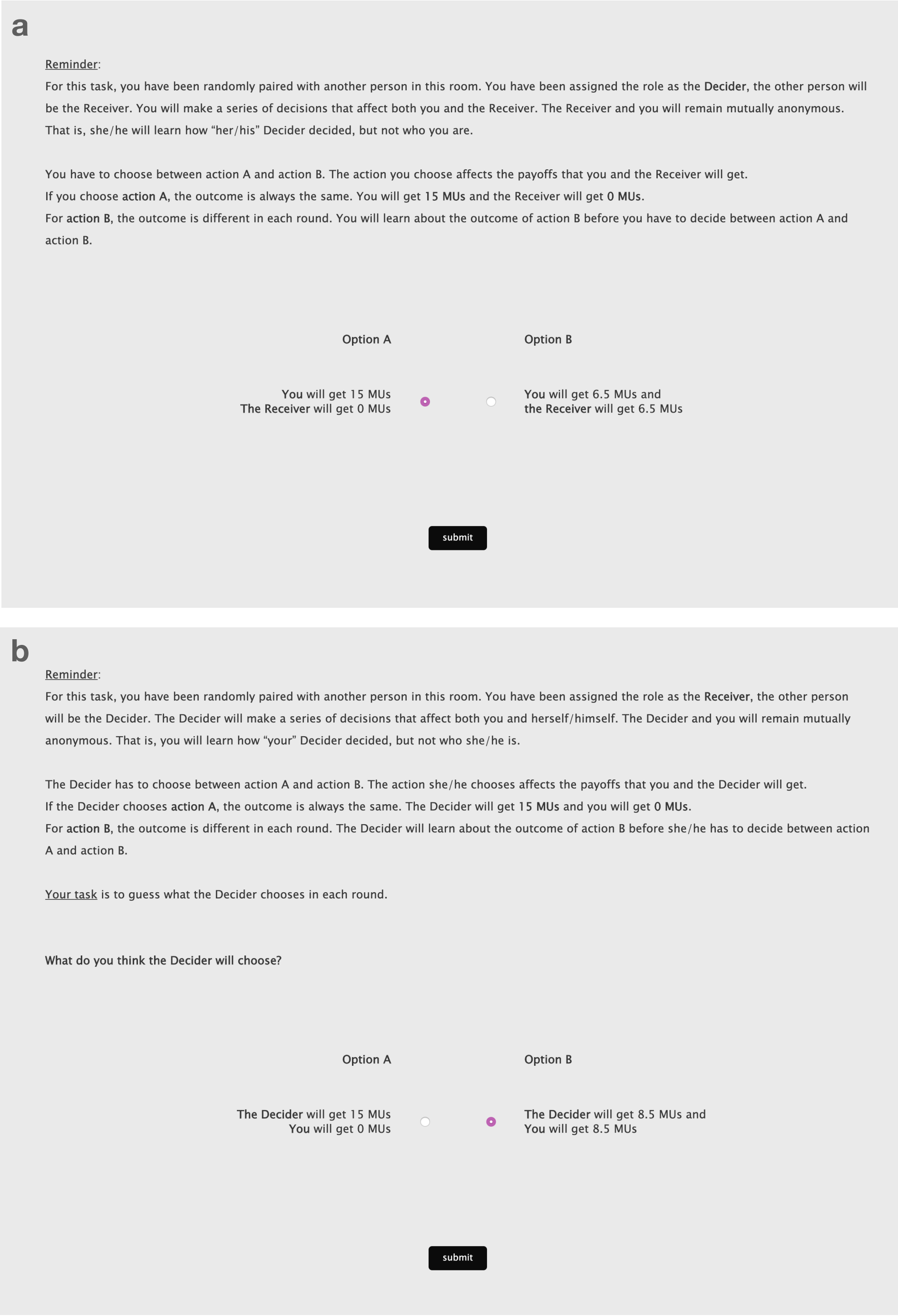

Regression models. As shown in Table S1 and S2, we fitted separate nested regression models to the risky helping choice data. In both data-sets a model that predicted risky helping based on a combination of risk and social preferences fitted the data better according to Likelihood Ratio tests compared to a model that tried to predict risky helping based on risk preferences or social preferences alone (Study 1: Model 1 vs. Model 3: Likelihood Ratio test, $p = 0.007$, Model 2 vs. Model 3: Likelihood Ratio test, $p < 0.001$; Study 2: Model 1 vs. Model 3: Likelihood Ratio test, $p < 0.001$, Model 2 vs. Model 3: Likelihood Ratio test, $p < 0.001$). In Study 1, an interaction model that assumes that the slope (in other words: the relationship) between risky helping and social (risk) preferences changes as a function of risk (social) preferences even outperformed the linear combination model (Study 1: Model 3 vs. Model 4: Likelihood Ratio test, $p = 0.020$). This, however, was not the case in Study 2 (Study 2: Model 3 vs. Model 4: Likelihood Ratio test, $p = 0.123$).

Table S1. Tobit regression models predicting risky helping based on risk and social preferences in Study 1.

	**(1)**	**(2)**	**(3)**	**(4)**
	B (p)	B (p)	B (p)	B (p)
Intercept	0.03 (0.550)	-0.01 (0.470)	-0.10 (0.007)	-0.04 (0.411)
risk preference	0.23 (0.110)		0.26 (0.006)	0.09 (0.448)
social preference		0.84 (< 0.001)	0.83 (< 0.001)	0.37 (0.061)
risk × social				1.28 (0.012)
AIC	77.19	-16.48	-21.81	-25.25
Log-Likelihood	-34.60	12.24	15.90	18.62

Table S2. Tobit regression models predicting risky helping based on risk and social preferences in Study 2.

	(1)	(2)	(3)	(4)
	B (p)	B (p)	B (p)	B (p)
Intercept	0.10 (0.003)	0.08 (< 0.001)	-0.03 (0.42)	-0.09 (0.101)
risk preference	0.66 (< 0.001)		0.31 (0.001)	0.48 (< 0.001)
social preference		0.73 (< 0.001)	0.73 (< 0.001)	0.92 (< 0.001)
risk × social				-0.55 (0.121)
AIC	-27.44	-113.19	-121.45	-121.83
Log-Likelihood	17.72	60.59	65.72	66.91

Monotonicity. We did not force participants to be consistent in their choice by, for example, letting them only choose a unique switching point across the choice menu, as sometimes done in lottery tasks to measure risk preferences. Instead participants made a choice for every single decision problem. This allowed us to identify violations of monotonicity, which can be interpreted as a sign for lower attention or engagement in the task. Table S3 shows the frequency of participants violating monotonicity across the different tasks in Study 1 and Study 2. We repeated the main regressions (shown in Table S1 and S2) only with participants that had a unique switching point (i.e. consistent choosers). Results are shown in Table S4 and S5. The general results pattern stayed consistent. Both, social and risk preferences significantly and independently predicted risky helping choices. As in the models on the full sample, a model that used, both, risk and social preferences as predictors outperformed simpler models that tried to predict risky helping decisions only with one preference as indicated by Likelihood Ratio tests (Study 1: Model 1 vs. Model 3:

Likelihood Ratio test, $p < 0.001$, Model 2 vs. Model 3: Likelihood Ratio test, $p = 0.045$; Study 2: Model 1 vs. Model 3: Likelihood Ratio test, $p < 0.001$, Model 2 vs. Model 3: Likelihood Ratio test, $p = 0.042$). The interaction model did not further increase model fit compared to the linear combination model based on a 5% significance level (Study 1: Model 3 vs. Model 4: Likelihood Ratio test, $p = 0.06$; Study 2: Model 3 vs. Model 4: Likelihood Ratio test, $p = 0.34$).

Table S3. Number of participants with consistent choice (i.e. unique switching point) and number and percentage of participants that violated monotonicity (i.e. multiple switching points) across tasks and studies.

		risk task	helping task	risky helping task
Study 1	# multiple	20	27	25
	# unique	126	119	121
	% multiple	13.7	18.5	17.1
Study 2	# multiple	37	42	36
	# unique	117	112	118
	% multiple	24.0	27.2	23.3

Table S4. Tobit regression models predicting risky helping based on risk and social preferences in Study 1 excluding participants with monotonicity violations.

	**(1)** B (p)	**(2)** B (p)	**(3)** B (p)	**(4)** B (p)
Intercept	-0.056 (0.461)	-0.054 (0.028)	-0.142 (0.006)	-0.074 (0.189)
risk preference	0.295 (0.137)		0.265 (0.041)	0.102 (0.476)
social preference		0.989 (< 0.001)	0.965 (< 0.001)	0.385 (0.192)
risk × social				1.456 (0.042)
AIC	69.78	7.05	5.02	3.36
Log-Likelihood	-30.89	0.47	2.49	4.32

Table S5. Tobit regression models predicting risky helping based on risk and social preferences in Study 2 excluding participants with monotonicity violations.

	**(1)** B (p)	**(2)** B (p)	**(3)** B (p)	**(4)** B (p)
Intercept	-0.003 (0.959)	0.044 (0.128)	-0.057 (0.317)	-0.127 (0.176)
risk preference	0.924 (< 0.001)		0.332 (0.038)	0.980 (< 0.001)
social preference		0.771 (< 0.001)	0.725 (< 0.001)	0.521 (0.042)
risk × social				-0.661 (0.344)
AIC	1.81	-49.45	-51.59	-50.49
Log-Likelihood	-30.89	0.47	2.49	4.32

Control regressions for Study 2. Table S6-S8 shows the influence of the drug treatment on risk-taking, helping, and risky helping behavior based on a stepwise inclusion of control variables and either fitting the model to the whole sample or a subsample of participants who guessed their drug-treatment incorrectly. Before the drug was administered, participants did not differ in attention, alertness, affective valence, and arousal. To control for mood and mood changes we reduced the dimensions of the mood questionnaire using factor analysis to avoid overspecification of the model (and resulting convergence problems due to multicollinearity of mood-items). Specifically, we performed exploratory factor analyses on the pre-treatment responses, the post-treatment responses, and the difference between pre- and post-treatment responses (i.e. mood change) of the 15-item mood questionnaire. In all three cases, a three-factor structure (determined by Horn's parallel analysis using Oblimin rotation) could parsimoniously capture the covariance-matrix of the 15-item mood items (accounting for 59%, 64%, and 59% of the variance of the pre-, post-, and mood-change responses, respectively). We labelled these three factors alertness (e.g. ‘muzzy vs. clearheaded’, ‘lethargic vs. energetic’, attentive vs. dreamy’), affective valence (e.g. ‘happy vs. sad’, ‘content vs. discontent’, ‘friendly vs. antagonistic’), and arousal (e.g. ‘excited vs. calm’, ‘tense vs. relaxed’, ‘troubled vs. tranquil’). We either controlled for differences in mood before the experiment, after the experiment, or changes in mood (i.e. difference between pre- and post-measure).

Table S6. Tobit regression models predicting risk-taking based on drug treatment, control variables, and excluding participants that guessed their drug treatment correctly.

	**(1)**	**(2)**	**(3)**	**(4)**	**(5)**	**(6)**	**(7)**	**(8)**	**(9)**	**(10)**	**(11)**
	B (p)	B (p)	B (p)	B (p)	B (p)	B (p)	B (p)	B (p)	B (p)	B (p)	B (p)
Intercept (placebo)	0.36<br>(0.000)	0.42<br>(0.000)	0.36<br>(0.004)	0.56<br>(0.000)	0.37<br>(0.004)	0.37<br>(0.003)	0.65<br>(0.000)	0.29<br>(0.023)	0.42<br>(0.002)	0.34<br>(0.003)	0.57<br>(0.000)
atomoxetine	0.02<br>(0.295)	0.02<br>(0.255)	0.03<br>(0.173)	0.04<br>(0.171)	0.03<br>(0.212)	0.01<br>(0.609)	0.01<br>(0.707)	0.03<br>(0.217)	0.05<br>(0.088)	0.02<br>(0.349)	0.02<br>(0.379)
methylphenidate	**0.05<br>(0.018)**	**0.05<br>(0.015)**	**0.06<br>(0.006)**	**0.14<br>(0.000)**	**0.06<br>(0.007)**	**0.05<br>(0.017)**	**0.13<br>(0.000)**	**0.06<br>(0.01)**	**0.14<br>(0.000)**	**0.05<br>(0.032)**	**0.12<br>(0.000)**
age	0.00<br>(0.511)	0.00<br>(0.454)	0.00<br>(0.329)	**-0.01<br>(0.017)**	0.00<br>(0.391)	0.00<br>(0.197)	**-0.01<br>(0.000)**	0.00<br>(0.690)	0.00<br>(0.128)	0.00<br>(0.240)	**-0.01<br>(0.002)**
sex (1 = male)	**0.04<br>(0.027)**	**0.05<br>(0.011)**	**0.05<br>(0.015)**	**0.07<br>(0.002)**	**0.05<br>(0.017)**	**0.05<br>(0.017)**	**0.08<br>(0.000)**	**0.05<br>(0.018)**	**0.07<br>(0.002)**	**0.05<br>(0.015)**	**0.08<br>(0.000)**
BMI		0.00<br>(0.438)	0.00<br>(0.344)	**-0.01<br>(0.010)**	0.00<br>(0.236)	0.00<br>(0.553)	**-0.01<br>(0.003)**	0.00<br>(0.526)	-0.01<br>(0.051)	0.00<br>(0.423)	**-0.01<br>(0.011)**
monotonicity violation					0.04<br>(0.094)	0.04<br>(0.064)	-0.01<br>(0.507)	0.03<br>(0.224)	0.00<br>(0.958)	0.03<br>(0.164)	0.00<br>(0.836)
alertness (pre)						0.00<br>(0.753)	-0.02<br>(0.227)				
affect (pre)						0.00<br>(0.965)	0.03<br>(0.119)				
arousal (pre)						-0.01<br>(0.637)	-0.01<br>(0.319)				
alertness (post)								**0.04<br>(0.008)**	**0.04<br>(0.018)**		
affect (post)								**0.04<br>(0.006)**	**0.04<br>(0.038)**		
arousal (post)								**0.04<br>(0.000)**	**0.04<br>(0.003)**		
alertness (change)										-0.02<br>(0.164)	-0.02<br>(0.295)
affect (change)										**-0.04<br>(0.007)**	-0.03<br>(0.157)
arousal (change)										**0.03<br>(0.011)**	0.02<br>(0.136)
medication dummies incl.	no	no	yes	yes	yes	yes	yes	yes	yes	yes	yes
correct guessers excl.	no	no	no	yes	no	no	yes	no	yes	no	yes

Note. BMI = Body Mass Index; medication dummies incl. = inclusion of dummy controls for contraceptive implant, contraceptive pill, ibuprofen (3/day), mefenamic acid, salbutamol; monotonicity violation dummy coded as 0 = unique switching-point, 1 = monotonicity violated; $p < 0.05$ highlighted in bold (omitted for the intercept).

Table S7 Tobit regression models predicting helping based on drug treatment, control variables, and excluding participants that guessed their drug treatment correctly.

	**(1)**	**(2)**	**(3)**	**(4)**	**(5)**	**(6)**	**(7)**	**(8)**	**(9)**	**(10)**	**(11)**
	B (p)	B (p)	B (p)	B (p)	B (p)	B (p)	B (p)	B (p)	B (p)	B (p)	B (p)
Intercept (placebo)	0.15 (0.227)	0.46 (0.016)	0.21 (0.415)	0.31 (0.351)	0.21 (0.448)	0.21 (0.428)	0.16 (0.552)	0.13 (0.62)	0.07 (0.837)	0.19 (0.488)	0.18 (0.593)
atomoxetine	0.00 (0.947)	-0.01 (0.891)	-0.02 (0.771)	0.00 (0.985)	-0.01 (0.823)	0.01 (0.911)	0.01 (0.848)	0.02 (0.711)	0.02 (0.774)	0.02 (0.683)	0.02 (0.765)
methylphenidate	0.02 (0.728)	0.03 (0.484)	0.04 (0.415)	0.10 (0.141)	0.05 (0.282)	0.06 (0.211)	**0.18 (0.006)**	0.05 (0.304)	0.11 (0.095)	0.05 (0.253)	**0.14 (0.035)**
age	0.01 (0.103)	0.01 (0.061)	0.01 (0.076)	0.00 (0.575)	0.01 (0.080)	0.01 (0.054)	0.01 (0.424)	**0.01 (0.040)**	0.01 (0.137)	**0.01 (0.047)**	0.01 (0.123)
sex (1 = male)	**-0.10 (0.012)**	-0.08 (0.060)	-0.07 (0.137)	-0.06 (0.278)	-0.08 (0.056)	-0.07 (0.085)	-0.07 (0.222)	-0.07 (0.056)	-0.06 (0.259)	-0.08 (0.052)	-0.07 (0.214)
BMI		**-0.02 (0.024)**	**-0.02 (0.007)**	**-0.02 (0.039)**	**-0.02 (0.006)**	**-0.02 (0.008)**	-0.01 (0.129)	**-0.02 (0.007)**	-0.02 (0.076)	**-0.02 (0.008)**	-0.02 (0.112)
monotonicity violation					0.06 (0.137)	0.08 (0.108)	0.09 (0.111)	0.06 (0.157)	0.09 (0.066)	0.07 (0.093)	**0.12 (0.022)**
alertness (pre)						0.00 (0.869)	0.00 (0.963)				
affect (pre)						0.06 (0.096)	**0.09 (0.036)**				
arousal (pre)						-0.03 (0.210)	**-0.07 (0.013)**				
alertness (post)								0.05 (0.093)	**0.10 (0.013)**		
affect (post)								**0.08 (0.015)**	**0.10 (0.016)**		
arousal (post)								0.02 (0.466)	0.01 (0.828)		
alertness (change)										-0.03 (0.249)	**-0.11 (0.007)**
affect (change)										-0.02 (0.469)	-0.04 (0.367)
arousal (change)										0.01 (0.615)	-0.01 (0.762)
medication dummies incl.	no	no	yes	yes	yes	yes	yes	yes	yes	yes	yes
correct guessers excl.	no	no	no	yes	no	no	yes	no	yes	no	yes

Note. BMI = Body Mass Index; medication dummies incl. = inclusion of dummy controls for contraceptive implant, contraceptive pill, ibuprofen (3/day), mefenamic acid, salbutamol; monotonicity violation dummy coded as 0 = unique switching point, 1 = monotonicity violated; $p < 0.05$ highlighted in bold (omitted for the intercept).

Table S8 Tobit regression models predicting risky helping based on drug treatment, control variables, and excluding participants that guessed their drug treatment correctly.

	(1)	(2)	(3)	(4)	(5)	(6)	(7)	(8)	(9)	(10)	(11)
	B (p)	B (p)	B (p)	B (p)	B (p)	B (p)	B (p)	B (p)	B (p)	B (p)	B (p)
Intercept (placebo)	0.19 (0.059)	0.43 (0.004)	0.26 (0.103)	0.69 (0.000)	0.26 (0.096)	0.26 (0.118)	0.43 (0.059)	0.17 (0.206)	0.23 (0.445)	0.20 (0.159)	0.34 (0.245)
atomoxetine	-0.01 (0.787)	-0.01 (0.819)	-0.01 (0.657)	0.00 (0.944)	-0.02 (0.544)	-0.02 (0.635)	0.01 (0.722)	-0.02 (0.477)	0.00 (0.937)	0.00 (0.990)	0.02 (0.793)
methylphenidate	**0.07 (0.047)**	**0.08 (0.026)**	**0.10 (0.001)**	**0.15 (0.001)**	**0.11 (0.001)**	**0.11 (0.003)**	**0.19 (0.000)**	**0.09 (0.015)**	0.11 (0.068)	**0.10 (0.004)**	**0.16 (0.044)**
age	0.00 (0.260)	0.01 (0.233)	0.00 (0.466)	**-0.01 (0.048)**	0.00 (0.571)	0.00 (0.451)	-0.01 (0.373)	0.00 (0.316)	0.00 (0.464)	0.00 (0.494)	0.00 (0.586)
sex (1 = male)	0.00 (0.901)	0.02 (0.521)	0.06 (0.075)	**0.14 (0.000)**	0.05 (0.153)	0.04 (0.200)	0.05 (0.297)	0.04 (0.127)	0.03 (0.489)	0.04 (0.154)	0.03 (0.527)
BMI		**-0.01 (0.030)**	**-0.01 (0.022)**	**-0.02 (0.002)**	**-0.01 (0.024)**	**-0.01 (0.031)**	-0.01 (0.084)	-0.01 (0.077)	-0.01 (0.159)	-0.01 (0.057)	-0.01 (0.271)
monotonicity violation					0.03 (0.321)	0.02 (0.504)	0.07 (0.082)	0.02 (0.465)	**0.11 (0.036)**	0.01 (0.805)	**0.13 (0.011)**
alertness (pre)						0.00 (0.852)	0.01 (0.871)				
affect (pre)						0.03 (0.244)	0.04 (0.225)				
arousal (pre)						-0.02 (0.207)	-0.04 (0.059)				
alertness (post)								0.02 (0.317)	**0.07 (0.049)**		
affect (post)								**0.05 (0.006)**	0.05 (0.215)		
arousal (post)								0.02 (0.141)	-0.01 (0.805)		
alertness (change)										-0.01 (0.692)	**-0.1 (0.021)**
affect (change)										-0.03 (0.166)	0.00 (0.929)
arousal (change)										0.01 (0.590)	-0.04 (0.230)
medication dummies incl.	no	no	yes	yes	yes	yes	yes	yes	yes	yes	yes
correct guessers excl.	no	no	no	yes	no	no	yes	no	yes	no	yes

Note. BMI = Body Mass Index; medication dummies incl. = inclusion of dummy controls for contraceptive implant, contraceptive pill, ibuprofen (3/day), mefenamic acid, salbutamol; monotonicity violation dummy coded as 0 = unique switching point, 1 = monotonicity violated; $p < 0.05$ highlighted in bold (omitted for the intercept).

Order effects. To relate risky helping decisions to risk and social preferences, each participant had to perform all three tasks. To control for potential order-effects, we randomized the order of tasks across participants. We further analyzed whether we found any statistical evidence that task-order influenced decision making. For that, we fitted Tobit regressions that tested whether switching points in each task varied as a function of (a) the position at which the task was performed (i.e. whether it was performed as the first, second, or third task in the experiment) and (b) the specific order in which the participant performed the task.

Table S9 shows the regression results, indicating to which degree task position influenced the observed switching points in the risk, helping, and risky helping task (separately for Study 1 and Study 2). We found that risk taking was influenced by task order in Study 1. According to the regression results, the later the task was performed in the experiment, the less risk a participant was willing to take (i.e. had a lower switching point). However, the task position coefficient was not significant in Study 2 and for all other tasks, task position was also not significantly related to the position of the switching point.

Table S10 shows the regression results, testing to which degree a specific order influenced the observed switching points in the risk, helping, and risky helping task (separately for Study 1 and Study 2). According to the model results, participants had a lower switching point in the risk task when the risk task followed the risky helping task and was proceeded by the helping task (RH R H order – compared to when risky helping and helping task were in the reversed position). Again, this order effect was not consistent across studies and we did not find any other significant order effects. We have to note that due to a programming mistake in the randomization procedure, we had no participants that performed the experiment in the R RH H order in Study 2 (i.e. starting with the risk task, then doing the risky helping task, and ending the experiment with the helping

task).

To further test whether our conclusions from the study stay robust when controlling for order effects, we repeated the analyses reported above (Table S1 & Table S2) and added order as additional predictors. The results are shown in Table S11 and Table S12. The reported effects did not substantially change and remained statistically significant. Further, as in the original analyses, a model that incorporated both risk and social preferences outperformed a model that only used one predictor (Study 1: Model 1 vs. Model 3: Likelihood Ratio test, $p = 0.02$; Model 2 vs. Model 3: Likelihood Ratio test, $p < 0.001$; Study 2: Model 1 vs. Model 3: Likelihood Ratio test, $p = 0.002$; Model 2 vs. Model 3: Likelihood Ratio test, $p < 0.001$).

We also re-fitted model 1-5 reported in Table S6-S8 and added the order control dummies. Also for these analyses, the reported effects did not substantially change. Methylphenidate had a significant effect on risk taking and risky helping, while we did not find significant associations between methylphenidate and helping rates and between atomoxetine and helping, risk taking, risky helping, as reported in the original analyses. We therefore conclude that controlling for order-effects did not change our study conclusions.

Table S9. Tobit regression models predicting switching points of the risk, helping, and risky helping task based on the position of the task (task position, coded as 0 = first task, 1 = second task, 2 = third task), separately fitted for Study 1 and Study 2.

	**Risk**		**helping**		**risky helping**	
	**Study 1**	**Study 2**	**Study 1**	**Study 2**	**Study 1**	**Study 2**
	B (p)	B (p)	B (p)	B (p)	B (p)	B (p)
Intercept	0.38 (<0.001)	0.38 (<0.001)	0.14 (<0.001)	0.29 (<0.001)	0.08 (0.006)	0.33 (<0.001)
Task position	**-0.04 (0.005)**	-0.02 (0.118)	-0.04 (0.21)	0.003 (0.893)	0.03 (0.14)	-0.01 (0.51)

Table S10. Tobit regression models predicting switching points of the risk, helping, and risky helping task based on the task order (R = risk task, RH = risky helping task, H = helping task) in which the experiment was performed, separately fitted to the data from Study 1 and Study 2.

	**Risk**		**helping**		**risky helping**	
	**Study 1**	**Study 2**	**Study 1**	**Study 2**	**Study 1**	**Study 2**
	B (p)	B (p)	B (p)	B (p)	B (p)	B (p)
Intercept (H R RH)	0.38 (<0.001)	0.37 (<0.001)	0.14 (0.036)	0.28 (<0.001)	0.18 (0.001)	0.31 (<0.001)
H RH R	-0.01 (0.862)	-0.03 (0.291)	0.02 (0.852)	0.05 (0.455)	-0.03 (0.696)	-0.03 (0.568)
R H RH	0.004 (0.907)	0.0002 (0.994)	-0.03 (0.735)	0.01 (0.884)	-0.07 (0.307)	0.002 (0.962)
R RH H	0.05 (0.261)	–	-0.08 (0.358)	–	-0.04 (0.593)	–
RH H R	-0.08 (0.053)	-0.06 (0.122)	-0.07 (0.405)	0.09 (0.207)	-0.08 (0.298)	-0.01 (0.917)
RH R H	**-0.08 (0.034)**	0.04 (0.193)	-0.06 (0.439)	0.04 (0.562)	-0.13 (0.055)	0.05 (0.343)

Table S11. Tobit regression models predicting risky helping based on risk and social preferences in Study 1.

	**(1)**	**(2)**	**(3)**	**(4)**
	B (p)	B (p)	B (p)	B (p)
Intercept	0.0009<br>(0.983)	0.12<br>(0.137)	-0.10<br>(0.103)	-0.05<br>(0.438)
risk preference		0.17<br>(0.260)	0.24<br>(0.016)	0.08<br>(0.494)
social preference	0.84<br>(<0.001)		0.84<br>(<0.001)	0.38<br>(0.041)
risk × social				1.27<br>(0.008)
H RH R order	-0.03<br>(0.642)	-0.03<br>(0.727)	-0.01<br>(0.831)	-0.0001<br>(0.998)
R H RH order	-0.02<br>(0.667)	-0.07<br>(0.299)	-0.01<br>(0.790)	-0.004<br>(0.927)
R RH H order	0.04<br>(0.426)	-0.05<br>(0.512)	0.04<br>(0.400)	0.05<br>(0.242)
RH H R order	0.01<br>(0.856)	-0.07<br>(0.390)	0.04<br>(0.450)	0.05<br>(0.311)
RH R H order	-0.06<br>(0.230)	-0.12<br>(0.092)	-0.03<br>(0.560)	-0.01<br>(0.786)
AIC	-13.21	83.67	-16.77	-20.57
Log-Likelihood	15.61	-32.84	18.38	21.29

Table S12. Tobit regression models predicting risky helping based on risk and social preferences in Study 2.

	**(1)**	**(2)**	**(3)**	**(4)**
	B (p)	B (p)	B (p)	B (p)
Intercept	0.07 (0.036)	0.11 (0.007)	-0.04 (0.452)	-0.09 (0.143)
risk preference		0.65 (<0.001)	0.30 (0.002)	0.46 (0.002)
social preference	0.73 (<0.001)		0.73 (<0.001)	0.90 (<0.001)
risk × social				-0.52 (0.142)
H RH R order	-0.03 (0.528)	-0.02 (0.595)	-0.02 (0.705)	-0.02 (0.623)
R H RH order	0.01 (0.764)	-0.01 (0.694)	0.01 (0.809)	0.003 (0.932)
RH H R order	0.02 (0.604)	-0.02 (0.576)	0.03 (0.442)	0.02 (0.589)
RH R H order	0.03 (0.415)	0.03 (0.460)	0.02 (0.621)	0.02 (0.687)
AIC	-107.69	-21.72	-114.98	-115.11
Log-Likelihood	61.85	18.86	66.49	67.56

Reaction times. In both studies, participants were not given any time limit to make their decisions and could perform the task at their own pace. We did, however, measured the time participants took to perform the task. Reaction times may provide further insights into the psychological mechanism of decision making when decisions are risky, influence the payoff of other participants, or both. We therefore analyzed whether switching points were related to the average time a participant took to make a decision (in Study 1 and Study 2) and whether drug administration influenced reaction times (Study 2).

We found that higher average time to decide was positively correlated with helping in both the helping and the risky helping task in Study 1 (helping: Spearman $r = 0.24$, $p = 0.003$, risky helping: Spearman $r = 0.33$, $p < 0.001$), meaning that participants that took longer to decide helped more. Average time to decide in the risk task was not significantly related to the switching point and hence inferred risk preferences of participants in the risk task (risk: Spearman $r = 0.06$, $p = 0.45$). In Study 2, higher average time to decide was also positively correlated with higher risky helping (risky helping: Spearman $r = 0.36$, $p < 0.001$) but not significantly related to helping (helping: Spearman $r = 0.12$, $p = 0.15$) and, compared to Study 1, time to decide was positively related with the switching point in the risk task, meaning that participants that took longer to decide were more risk seeking (risk: Spearman $r = 0.19$, $p = 0.02$). However, the results in the helping and risky helping tasks may be confounded by purely selfish participants that decided to never help a priori and therefore made faster decisions by always choosing Option A without much thinking. And, indeed, when we calculated correlations by excluding participants that never helped in the helping and risky helping task, the correlations became insignificant in Study 1 (helping: Spearman $r = 0.17$, $p = 0.11$, risky helping: Spearman $r = 0.10$, $p = 0.34$). The association of reaction time and risky helping however remained significant in Study 2 (helping: Spearman $r = 0.03$, $p = 0.74$, risky

helping: Spearman $r = 0.32$, $p < 0.001$).

For Study 2, we further tested whether average reaction time across all three tasks and for each task separately was influenced by the drug treatment using linear regression models in which we also controlled for age and sex. Overall reaction time was descriptively higher when participants were given methylphenidate (compared to placebo, b = 465.49 msec, $p = 0.07$), while we did not observe a difference between placebo and atomoxetine (b = 250.05 msec, $p = 0.31$). Comparing each task separately, we only found one significant difference in the helping task. Participants given methylphenidate took 656.30 msec longer to decide according to the model, which was significantly more compared to placebo (b = 656.30 msec, $p = 0.03$). All other comparisons were above the statistical threshold of $p > 0.05$.

Taken together, these results seem to tentatively suggest that more deliberation was associated with more helping and risky helping and that methylphenidate increased deliberation time. However, the results are not very strong, possibly confounded by reaction times of purely selfish individuals, and not consistent across studies. Since the study was not designed to test reaction time or deliberation in association with risky helping, future studies are needed to directly test whether deliberation vs. impulsive decision making is associated with different degrees of helping or risky helping, by, for example, introducing time pressure or forcing participants to deliberate about their decision before they are allowed to choose their preferred option.

Additional results. While not the main aim of this investigation, Study 1 also allowed us to test how receivers attributed helping decisions under risk based on their estimated social and risk preferences of their deciders, since they had to guess each decision of the decider. Receivers did not expect more helping with (Figure S10c, Mann Whitney U-test, $p = 0.18$) or without risk (Figure

S10b, Mann Whitney U-test, p = 0.35, see also Figure S10). They did however, estimate the deciders to be more risk-seeking than they actually were (Figure S10a, Mann Whitney U-test, $p < 0.001$). Importantly, receivers' risky helping expectations were mainly driven by their estimated social preferences of their decider (Tobit regression, social preference estimate = 0.96, p = 0.01), but not by their estimated risk preferences of their decider (Tobit regression, risk preference estimate = -0.21, p = 0.18), or the interaction of expected risk and social preferences (Tobit regression, risk preferences × social preferences estimate = -0.19, p = 0.80). Put differently, receivers did not consider that both social and risk preferences condition the extent of helping under risk and instead misattributed risky helping (or the lack thereof) to social preferences alone.

Figure S10. *Receiver's expectation vs. decider's actual helping behavior.* Average expected switching points of receivers (grey) versus actual switching points of deciders (blue) across (a) risk, (b) helping, and (c) risky helping task.

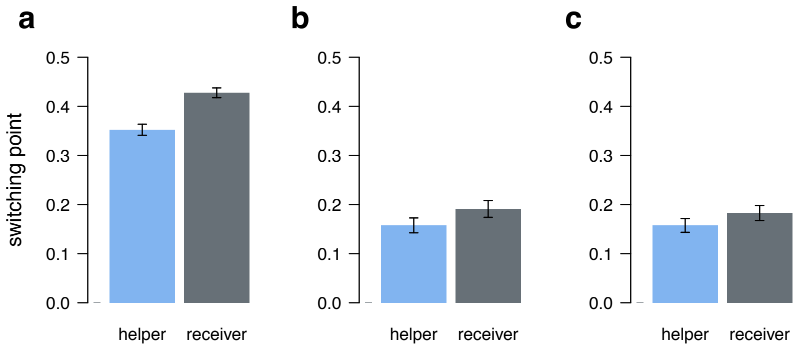

Supplement: Supplemental material [file EMS160406-supplement-Supplemental_material.pdf]
